# Crop-type-driven changes in polyphenols regulate soil nutrient availability and soil microbiota

**DOI:** 10.3389/fmicb.2022.964039

**Published:** 2022-08-24

**Authors:** Dongmei Fan, Zhumeng Zhao, Yu Wang, Junhui Ma, Xiaochang Wang

**Affiliations:** ^1^Department of Tea Science, College of Agriculture and Biotechnology, Zhejiang University, Hangzhou, China; ^2^Hainan Institute, Zhejiang University, Sanya, China; ^3^Administration of Agriculture and Rural Affairs of Lishui, Lishui, China

**Keywords:** continuous cropping, rotation, polyphenols, soil microorganism, functional profiles, tea

## Abstract

Crop rotation is a typical agronomic practice to mitigate soil deterioration caused by continuous cropping. However, the mechanisms of soil biotic and abiotic factors in response to different cropping patterns in acidic and polyphenol-rich tea nurseries remain unclear. In this study, the composition and function of microbial communities were comparatively investigated in soils of tea seedlings continuously planted for 2 years (AC: autumn-cutting; SC: summer-cutting) and in soils rotation with strawberries alternately for 3 years (AR: autumn-cutting). The results showed that AR significantly improved the survival of tea seedlings but greatly reduced the contents of soil polyphenols. The lower soil polyphenol levels in AR were associated with the decline of nutrients (SOC, TN, Olsen-P) availability, which stimulates the proliferation of nutrient cycling-related bacteria and mixed-trophic fungi, endophytic fungi and ectomycorrhizal fungi, thus further satisfying the nutrient requirements of tea seedlings. Moreover, lower levels of polyphenols facilitated the growth of plant beneficial microorganisms (*Bacillus*, *Mortierella*, etc.) and suppressed pathogenic fungi (*Pseudopestalotiopsis*, etc.), creating a more balanced microbial community that is beneficial to plant health. Our study broadens the understanding of the ecological role of plant secondary metabolites and provides new insights into the sustainability of tea breeding.

## Introduction

Due to the high interest in tea [*Camellia sinensis (L.) O. Kuntze.*], tea plantation area is continually and rapidly expanding since the early 21st century. China has the largest tea plantation area worldwide, with 2.93 million hectares, accounting for 61% of the global tea planting area. The continuous expansion of tea plantations has increased the demand for tea seedlings. Single-node cutting technology is the most widely used method of tea propagation because it can maintain the desirable character of the female parent and shorten the breeding time to about 1 year (Jiang, 2011). Due to limited land resources and climatic constraints of tea seedlings, continuous cropping of tea seedlings is widely adopted in southern China, and the planting density is extremely high, with 3 ∼ 3.75 million tea seedlings per hectare. However, continuous tea seedling cropping results in severe soil acidification and plant diseases, which significantly depresses yield and quality (Ye et al., 2016). In production, chemical disinfection and annual subsoil mulching (covering the nursery soil surface with a 5 cm thick yellow subsoil) are measures frequently used to prevent continuous cropping obstacles for tea seedlings. But these two measures are costly and ecological threatening, and finding a better solution is essential to promote the sustainable development of tea propagation (Wang et al., 2015).

Crop rotation is a practical approach to alleviate continuous cropping obstacles from abiotic and biotic aspects (Liu et al., 2020; Lyu et al., 2020). In contrast to continuous monocultures, crop rotation can change litter quality and the composition of the root exudate by increasing the diversity of plants, thus providing a wider variety of residual carbon substrates in the soil and supporting the growth of different microorganisms. Some studies have shown that crop rotation can increase soil nutrient contents and decrease the incidence of soil-borne diseases (Lupwayi et al., 1998; Liu et al., 2019), thereby increasing crop yields and recovery soil health (Arriaga et al., 2017). For instance, apple seedlings-shallot rotation significantly increased the biomass of apple seedlings and SOC contents compared with the continuously cropped apple seedlings (Lv, 2014). As a result of a 5-year rotation of soybean and maize, an increase in plant growth-promoting microbes *Bradyrhizobium* and *Gemmatimonas*, *Mortierella* and *Paecilomyces*, and a decrease in potential pathogenic fungi *Fusarium* was found compared to 3 and 5 years’ continuous soybean cropping (Liu et al., 2020). Studies have shown that microbial diversity increases with rotating crop systems (Lupwayi et al., 1998). In comparison, others have found that changes in cropping systems have no significant effect on soil microbial diversity (Navarro-Noya et al., 2013). These inconsistent findings suggest that there are discrepancies in the effects of cropping type on soil microbial communities in different ecosystems.

Distinct from other agricultural systems, tea plantations are featured by severe acidification and polyphenols accumulations. Previous studies revealed that large amounts of polyphenols entered and accumulated in the soils of tea plantations through litter decomposition and root exudation (Fan et al., 2016), far more than in forest (Tharayil et al., 2013). Polyphenols are aromatic benzene ring compounds with one or more hydroxyl groups, their unique chemical structures allow polyphenols participate in a series of ecological processes, such as litter decomposition, humification (Northup et al., 1998), plant defense (Lattanzino et al., 2006), and nutrient cycling (Vitousek, 2000). Furthermore, polyphenols are also very typical allelochemical substances causing plant self-toxicity. In the soil of continuously cropped strawberries and cucumbers, polyphenols are considered the leading cause of continuous cropping obstacles (Ma et al., 2005; Tian, 2015). Besides, polyphenols also regulate microbial community composition and structure because they serve as a carbon source for certain microorganisms and toxic substances to inhibit some microbes’ proliferation (Schimel et al., 1998; Vitousek, 2000). Thus, soil polyphenols might be the key to clarifying the continuous cropping obstacles in tea nurseries.

Previous studies have shown that long-term tea monoculture significantly shifts soil bacterial and fungal communities, including microbial diversity, composition, and structure (Wang, 2015), the beneficial microbes such as *Bacillus*, *Sphingomonas*, and *Prevotella* were also significantly decreased in their relative abundance (Arafat et al., 2020). Nitrogen-fixing bacteria were found to be more prevalent in younger tea gardens than in the older ones, both in terms of species and quantity of bacteria (Tian, 2000). However, the microbial succession patterns observed in consecutive monoculture tea plantations and other continuous cropping systems are not fully applicable to continuously cropped tea nurseries. We therefore investigated soil polyphenol dynamics as well as soil bacterial and fungal composition and function in soils from the 2-year continuous cropping of tea seedlings cutting in different seasons and soils from the 3-year rotation of tea seedling-strawberry-tea seedling in Southeast China. The main purposes of this study were (1) to reveal the variations in soil physicochemical properties especially soil polyphenols under different crop types; (2) to reveal the response of diversity, composition and function of bacterial and fungal communities; (3) to investigate how polyphenols influence microbial communities and functions; (4) and to explore the intrinsic mechanisms of continuous cropping obstacles in acidic and polyphenol-rich ecosystems. The findings of the study will help us gain insight into the ecological role of secondary metabolites in the terrestrial ecosystem, and help guide the sustainable development of tea plantations and tea nurseries.

## Materials and methods

### Experimental site and design

The study site was located at a family farm in Dagangtou, Lishui, Zhejiang Province (28°28′ N, 119°76′ E), Southern China. A subtropical monsoon climate is prevailing at the experimental site, with an annual average temperature of 18.4°C and an annual precipitation of 1406.0 mm. The local soil is classified as Ultisols (United States Department of Agriculture [USDA] Natural Resources Conservation Services, 1998 classification). Before the experiment, the substrates (0–20 cm) in 2017 contained soil organic matter (SOC) 15.7 g kg^–1^, total nitrogen (TN) 2.04 g kg^–1^, available phosphorus (Olsen-P) 54.72 mg kg^–1^, available potassium (AK) 43.11 mg kg^–1^, and pH 4.39.

Since significant continuous cropping obstacles can occur within the second year of continuous cropping in tea nurseries, short-term trials were conducted from 2017 to 2019 to assess the influence of crop types. The experiment included three treatments: 2 years of continuous cropping with tea seedlings planted in summer (July) and autumn (November), which were hereafter referred to as the SC and AC treatments, respectively; 2 years’ rotation of strawberries-tea seedlings, the soil of the rotation treatment was the nursery soil where tea seedlings had been planted for 1 year; strawberries were planted in September 2017, and tea seedlings were cultivated in November 2018 after the strawberries were harvested in June 2018, referred to as AR treatment. The tea variety used in the experiment was *Longjing 43*, and the strawberry variety was *Hongyan*. Each treatment was 45 m^2^ in area with four replicated plots (1.5 m × 7.5 m × 4). The planting density of tea seedlings was a 2.4–2.7 million per hectare. Compound fertilizer (N-P_2_O_5_-K_2_O:15-15-15) was applied at a fertilization rate of 300 kg ha^–1^ during the tea seedlings plantation. Potassium dihydrogen phosphate (600 kg ha^–1^) and rapeseed cake (750 kg ha^–1^) were fertilized during strawberry plantation. Soil samples were collected in September 2019, twelve topsoil samples (0–10 cm depth) were randomly selected and completely mixed into one composite sample at each plot. The mixed soil samples were transported to the laboratory on ice immediately, then passed through a 2-mm sieve after removing the fine roots and visible particles. Portions of soil samples (about 120 g each) were stored at −80°C until the molecular analysis. The remaining samples were air-dried and screened for 60 mesh for subsequent analysis of physicochemical properties.

### The survival percentage of tea seedlings and soil physicochemical properties

The random survey method was adopted to assess the survival rate of tea seedlings. Four 1 m × 1 m quadrats were randomly selected in each treatment, and tea seedlings were collected to obtain the total number of the surviving tea seedlings “m.”

The survival rate of tea seedlings in different treatments was calculated by the following equation:


Survival percentage(%)=[(m/4)×45/(1×1)]/n×100%

Where *n* represents the number of the original planted tea seedlings for each treatment, with a range of 10800–12150; 45 was the area of each treatment, and 1 × 1 was the area of each survey quadrat; 4 represents the number of survey quadrats in each treatment.

Soil pH was measured by a glass electrode method (2.5:1 water/soil ratio). Nitrate (NO_3_^–^) and ammonium (NH_4_^+^) were extracted using 2 M KCl and determined on a flow injection autoanalyzer (FLA star 5000 Analyzer, Foss, Denmark). SOC was measured by the standard potassium dichromate digestion. TN was measured by the Kjeldahl method (Bremner, 1960). AK was detected by ammonium acetate extraction and flame photometry, while Olsen-P was detected using sodium bicarbonate extraction and molybdenum-antinomy colorimetry (Olsen, 1954). Soil polyphenols, including low- and high-molecular-weight ones (hereafter abbreviated as Lp and Hp, respectively) were determined using Folin-Ciocalteu method (Box, 1983), as modified by Kanerva et al. (2008). Briefly, 0.5 g of soil sample was extracted with ultra-pure water, and the high-molecular-weight polyphenols (about >0.5 kDa) were precipitated with 1.0 g of casein (Haslam and Cai, 1994). Total soluble polyphenols were measured based on the formation of the colored complex between phenolics and the alkaline Folin-Ciocalteu reagent (Suominen et al., 2003). We calculated the proportion of high-molecular-weight polyphenols by subtracting the results of the untreated samples from those of the casein-treated samples. According to the detection method for tea polyphenols, gallic acids were adopted as the standard in the current study, and the absorbance was measured at 760 nm (Zhou et al., 2009).

### Soil DNA extraction

An aliquot of 0.5 g of frozen soil was used to extract soil DNA using the QIAGEN PowerSoil kit (Germany). The quality and concentration of DNA were evaluated using a NanoDrop 2000 spectrophotometer (Thermo Fisher Scientific, United States) and agarose gel electrophoresis (Supplementary Figure 1). The obtained DNA was then stored at −20°C for subsequent processing.

### High throughput sequencing

A next-generation sequencing library was prepared and Illumina MiSeq sequencing was conducted at Majorbio (Shanghai, China). The bacteria gene V4 region and the fungal gene ITS1 region were amplified using primers 515modF/806RmodR (Sampson et al., 2016; Walters et al., 2016) and 1337F/2043R (Zhang L. et al., 2016; Zhang W. et al., 2016), respectively. All the DNA samples were amplified in triplicate in a thermocycler PCR system (GeneAmp 9700, ABI, United States). Triplicate PCR amplicons were mixed, followed by electrophoretic detection in a 2% (w/v) agarose gel and purified using the AxyPrep DNA Gel Extraction Kit (Axygen, United States), further quantified using QuantiFluor-ST (Promega, United States) in accordance with the manufacturer’s protocol. The purified PCR amplicons were sequenced on the Illumina MiSeq (300-bp paired-end reads) platform (Illumina Inc., United States). Under the project accession number PRJNA738594, the raw reads were submitted to the NCBI Sequence Read Archive.

The raw fastq files were quality-filtered by Trimmomatic and merged by FLASH (version 1.2.11) based on the criteria listed below: (1) Over a 50-bp sliding window, the reads were truncated at any site with an average quality score below 20; (2) Sequences with overlap more than 10 bp were merged based on their overlap with a maximum mismatch of 2 bp; (3) Based on the barcodes (exact matching) and the primers (allowing for two nucleotide mismatches), the sequences for each sample were split and reads containing ambiguous bases were eliminated. Sequence analysis was carried out using QIIME software (Caporaso et al., 2010). Similar sequences were clustered into operational taxonomic units (OTUs), based on 97% similarity using the UPARSE pipeline (ver. 7.1). Bacteria were identified taxonomically (99% similarity) by using the 16S rRNA SILVA database, and fungi were identified by using the UNITE database. In order to avoid differences in biomass among samples, sample sequences were normalized based on the minimum sequence number to obtain standardized data for subsequent statistical analysis.

### Statistical analysis

The variations in soil physicochemical properties, the survival percentage of tea seedlings, the relative abundance of bacterial and fungal taxa, the bacterial and fungal alpha-diversity indexes, and the bacterial and fungal functions were all tested by one-way ANOVA. The relationship between soil physicochemical properties and alpha-diversity, microbial taxa, bacterial function, and fungal functional guilds was achieved by Spearman or Pearson correlation coefficient using SPSS statistical software (SPSS 25.0, United States). The α-diversity of OTU richness and the Shannon index was calculated using Mothur^1^ to assess microbial diversity and abundance. Spearman’s correlation coefficients were analyzed between soil physicochemical properties and α-diversity index and community composition obtained with SPSS at *P* < 0.05. Based on Bray-Curtis distance, the analysis of principal coordinates (PCoA) and similarities (ANOSIM analysis) of microbial communities in tea nursery soils was performed with the corresponding function in the “vegan” package in the R package to determine the effect of crop type changes. Effects of cropping system and cropping time on microbial communities were also analyzed with the PERMANOVA (permutational analysis of variance) using “vegan” package in R. The linear regression model was used to investigate the correlations between the concentrations of soil polyphenols (Hp and Lp) and soil TN, SOC, NH_4_^+^, NO_3_^–^, Olsen-P, AK, and pH values.

Redundancy analysis (RDA) plots were also generated in the “vegan” package in the R package. Functional profiles of bacterial and fungal taxa were carried out using the FAPROTAX and FUNGuild databases, respectively. Heatmaps were created using GraphPad Prism software (GraphPad Software 8.0, United States) to reveal the relationship between microbial genera and microbial community functions and soil physicochemical properties, and to determine the differences in predicted bacterial functions in different soil samples. To quantify the impacts of soil polyphenols on soil nutrient, pH, and changes in bacterial and fungal communities, a structural equation model (SEM) was constructed using Amos (version 26.0). In order to fit the covariance matrix to the model, a robust maximum likelihood estimation method was used. Chi-square (χ^2^), degree of freedom, and *P* values were used to assess the fitness of the SEM model. All non-significant parameters of the original model were excluded in order to obtain the most parsimonious and best fitting model.

## Results

### Soil physicochemical properties and the survival percentage of tea seedlings

The highest survival percentage of tea seedlings was presented in AR (85.16%), which is significantly higher than that in AC and SC; and the lowest survival percentage was 14.27% in AC (Table 1). The contents of SOC, TN, Lp, Hp, and Olsen-P differed among the tea nurseries and were significantly higher in AC and SC than in AR. The AC site had the lowest soil pH value, which differed significantly from the other two treatments. The AR soil had the highest AK, NO_3_^–^, and NH_4_^+^ contents than that in SC and AC.

**TABLE 1:** Soil parameters in tea nurseries under different crop types.

Soil parameters	SC	AC	AR
Survival percentage of tea seedlings (%)	74.02 ± 0.60b	14.27 ± 0.15a	85.16 ± 0.76c
pH	4.36 ± 0.02b	4.03 ± 0.01a	4.38 ± 0.01b
SOC (g kg^–1^)	20.77 ± 0.00b	21.52 ± 0.00c	13.46 ± 0.00a
TN (g kg^–1^)	2.26 ± 0.04b	2.38 ± 0.00c	1.94 ± 0.01a
Lp (mg kg^–1^)	74.77 ± 4.70b	141.89 ± 13.13c	34.57 ± 3.93a
Hp (mg kg^–1^)	1501.98 ± 77.83b	1636.64 ± 39.01c	1335.16 ± 44.15a
Olsen-P (mg kg^–1^)	192.53 ± 8.92c	175.75 ± 3.68b	72.94 ± 2.08a
AK (mg kg^–1^)	107.58 ± 2.03a	154.99 ± 2.47b	186.11 ± 1.67c
NO_3_^–^ (mg kg^–1^)	14.82 ± 0.01a	33.51 ± 0.01b	53.46 ± 0.00c
NH_4_^+^ (mg kg^–1^)	10.79 ± 0.01a	15.80 ± 0.00b	39.00 ± 0.01c

Means are presented with standard deviation (n = 4), different letters represent significant differences among the three treatments at the *P* < 0.05 level. Lp, low-molecular-weight polyphenols; Hp, high-molecular-weight polyphenols; Olsen-P, soil available phosphorus; SOC, soil organic carbon; TN, soil total nitrogen; AK, soil available potassium; NO_3_^–^, soil nitrate; NH_4_^+^, soil ammonium.

### Bacterial and fungal community diversities in tea nurseries

**FIGURE 1:**
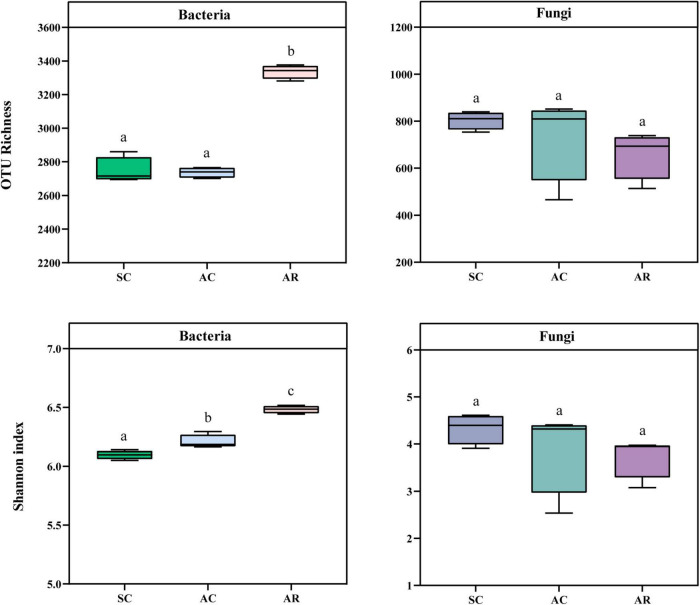
Comparison of the α-diversity indices of the bacterial and fungal community in tea nursery soils under three different crop types. Error bars represent the standard deviation (*n* = 4), and different lower-case letters represent significant differences (*P* < 0.05).

Since the rarefaction curves reached saturation, indicating that the number of sequences was sufficient to assess the microbial diversity in this study (Supplementary Figure 3). The bacterial OTU richness and Shannon index in the SC and AC treatments were significantly lower than in the AR treatment (Figure 1). In contrast, no difference in the fungal OTU richness and Shannon index was observed among the different sites (Figure 1). The bacterial OTU richness was positively correlated with soil pH, AK, NO_3_^–^, and NH_4_^+^ but negatively related to soil Lp, Olsen-P, SOC, and TN; the Shannon index of bacteria was positively correlated with soil AK, NO_3_^–^, and NH_4_^+^ contents, but negatively correlated with soil Lp, Hp, and Olsen-P concentrations (Supplementary Table 1). None of the soil physicochemical parameters was significantly associated with fungal diversity indices (Supplementary Table 1).

### Soil microbial community composition at taxonomic level

**FIGURE 2:**
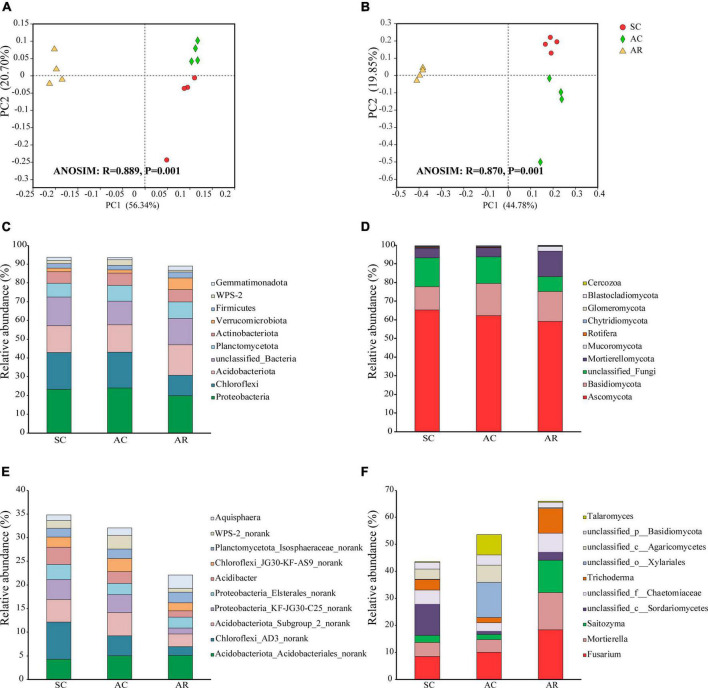
General patterns of microbial beta-diversity and the dominant bacteria and fungi in tea nursery soils under different planting patterns. PCoA showed the structure of the microbial communities of bacteria **(A)** and fungi **(B)**. Similarity values among the three sites were examined by the ANOSIM test, which is shown in each plot. The top 10 most abundant bacterial phyla **(C)**, genera **(E)**, and fungal phyla **(D)**, genera **(F)** in soil samples from the SC, AC, and AR sites.

From the 12 soil samples in this study, 849,879 bacterial quality sequences and 433,992 fungal quality sequences were obtained and clustered into 6026 and 2411 OTUs, respectively. The bacterial community was comprised of 41 phyla, including 140 classes, 311 orders, and 744 genera. *Proteobacteria* (22.3% of the sequences) was the most abundant bacterial phylum across all samples, followed by *Chloroflexi* (16.6% of the sequences), and *Acidobacteriota* (15.0% of the sequences; Figure 2C). The relative abundance of some bacterial phyla differed significantly under different treatments. The relative abundances of phyla *Verrucomicrobiota*, *Myxococcota*, *Desulfobacterota*, and *Methylomirabilota* were the highest in the AR site, whereas the relative abundances of *Chloroflexi* and *WPS-2* were higher in the SC and AC sites (Supplementary Table 2). *Acidobacteria*, *Gammaproteobacteria*, and *Alphaproteobacteria* were the most dominant bacteria at the class level across all soil samples (Supplementary Table 3). In the AC and AR treatments, Acidobacteriota_*Acidobacteriales_norank* was the most abundant bacterial genus, while Chloroflexi_*AD3_norank* dominated in the SC treatment at the genus level (Figure 2E and Supplementary Table 4). Among the 59 bacterial genera with a relative abundance of more than 0.5%, 43 showed significant variations among the treatments (Supplementary Table 4). The influence of cutting time on the bacterial community was relatively weaker than the cropping system (Supplementary Table 8): only the relative abundance of two phyla (*WPS-2* and *Cyanobacteria*), five classes (*AD3*, *Anaerolineae*, *WPS-2_norank*, *Holophagae*, and *Cyanobacteria*) and twelve genera (*Chloroflexi_AD3_norank*, *Acidibacter*, *Chloroflexi_RBG-13-54-9_norank, Chujaibacter*, etc.) were significantly different in the SC and AC treatments (Supplementary Tables 2–4).

The fungal OTUs were classified into 16 phyla, including 41 classes, 90 orders, and 324 genera. *Ascomycota* (62.2% of the sequences) was the predominant fungal phylum across all soil samples in this study, followed by *Basidiomycota* (15.2% of the sequences; Figure 2D). The relative abundances of phyla *Mortierellomycota* and *Mucoromycota*, and classes *Mortierellomycetes*, *Tremellomycetes*, *Pezizomycetes*, and *Mucoromycetes*, significantly increased in the AR treatment compared with the SC and AC treatments (Supplementary Tables 5, 6). *Unclassified_c_Sordariomycetes* (belonging to *Ascomycota*; 11.5% of the sequences), *unclassified_o__Xylariales* (belonging to *Ascomycota*; 13.0% of the sequences), and *Fusarium* (belonging to *Ascomycota*; 18.5% of the sequences) were the most abundant fungal genus in the SC, AC, and AR treatments, respectively (Figure 2F and Supplementary Table 7). The relative abundances of nine genera (*Mortierella*, *Trichoderma*, etc.) significantly increased, while two genera (*Neurospora* and *Westerdykella*) dramatically decreased under the rotational treatment AR compared to the two continuous cropping treatments SC and AC. Consistent with bacterial communities, the cropping system had greater impact on fungal community composition compared to cropping time: only one fungal class (*Pezizomycetes*) and seven genera (*Simplicillium*, *unclassified_f_Nectriaceae*, etc.) differed significantly in relative abundance between the AC and SC treatments (Supplementary Tables 7, 8).

### Soil bacterial and fungal community structures

At the OTU level, the three tea nurseries under different crop types shared 40.74% of the bacterial OTUs and 17.30% of the fungal OTUs (Supplementary Figure 2). SC and AC treatments shared the highest number of fungal OTUs (463 OTUs), while AR had the highest number of unique bacterial and fungal OTUs (Supplementary Figures 1A,B). The PCoA analysis showed that soil samples selected from different crop types formed distinct bacterial and fungal community structures (Figures 2A,B). Moreover, soil samples from SC and AC were far from that from AR along the abscissa axis. This result was further confirmed by ANOSIM tests, suggesting the remarkable changes in bacterial and fungal community structures induced by different crop types.

**FIGURE 3:**
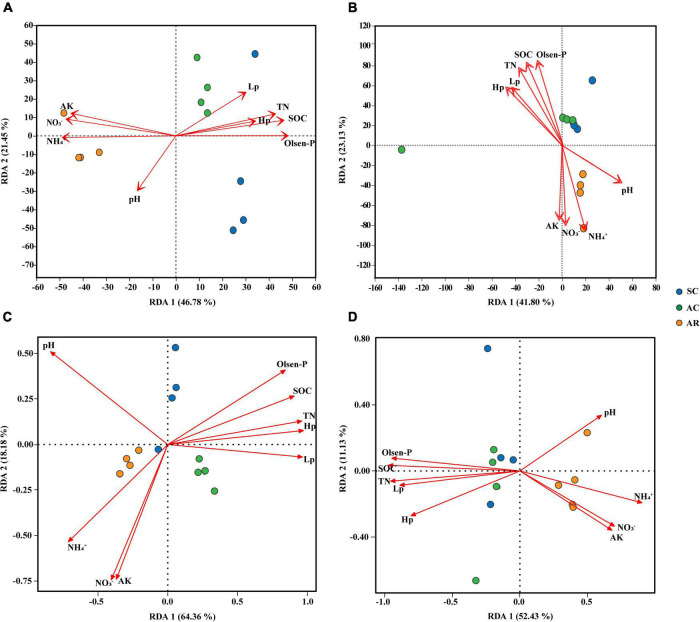
Redundancy analysis (RDA) reveals the relationships between soil properties and soil microbial community structures and functions among soil samples from tea nurseries under different crop types. Taxonomic analysis of bacteria **(A)** and fungi **(B)** using relative abundance based on 16S rRNA and ITS1 gene at the genera level. Functionality analysis of bacteria **(C)** and fungi **(D)** based on FARPROTAX and FUNGuild database. The values of axis 1 and 2 are the proportions explained by the corresponding axis. Arrows indicate correlations between soil chemical properties and microbial taxonomic and functional profiles.

Redundancy analysis (RDA) was performed to evaluate the impact of soil physicochemical properties on microbial community composition and function (Figures 3A–D). The first and second axis collectively contributed over 68 and 64% of the total variation in the bacterial and fungal community composition, respectively (Figures 3A,B). In addition to the crop type, soil properties also significantly influenced the community composition of bacteria and fungi: for bacterial communities, the contribution of soil properties was NH_4_^+^ > Olsen-P > NO_3_^–^ > SOC > AK > TN > Lp in order; for fungi, the contribution was SOC > Olsen-P > NH_4_^+^ > TN > NO_3_^–^ > AK > Hp > Lp (Figures 3A,B and Supplementary Table 9).

### Bacterial and fungal community functionalities and functional structure

**FIGURE 4:**
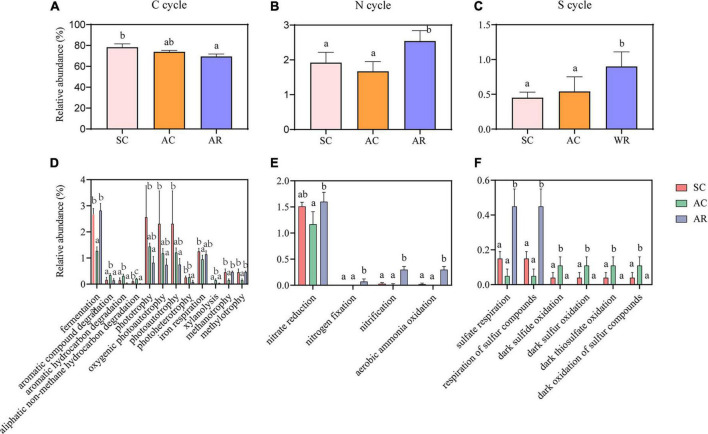
Differences in the relative abundance of nutrient cycling-related functions dominated by bacterial communities, namely C cycle **(A)**, N cycle **(B)**, S cycle **(C)** and the functional groups with significant variations involved in C **(D)**, N **(E)**, and S **(F)** cycles. Different letters above the column represent significant differences among the three tea nurseries (*P* < 0.05).

FAPROTAX database was adopted to predict the potential functions of soil bacterial communities that contribute to C, N, and S cycling (Louca et al., 2016). Fifty functional groups were obtained in total, and these functional floras containing 743 OTUs accounted for 12.32% of total OTUs. C cycle-related functional groups dominated in tea nurseries, accounting for 69.47–78.26% of all functions (Figure 4A). AR had the highest number of sequences related to nitrification, aerobic ammonia oxidation, nitrogen fixation, sulfate respiration, and respiration of sulfur compounds involved in the N and S cycles compared with the SC and AC (Figures 4E,F). The functional groups: photoheterotrophy and aliphatic non-methane hydrocarbon degradation involved in the C cycle presented higher abundance in SC and AC compared with AR (Figure 4D). The cutting season also had a significant influence on the bacterial functional groups: AC treatment showed the higher proportions of sequences affiliated with the decomposition of aromatic compounds, aromatic hydrocarbons, aliphatic non-methane hydrocarbons, and xylanolysis for the C cycle, as well as dark sulfide oxidation, dark sulfur oxidation, dark oxidation of sulfur compounds, and dark thiosulfate oxidation for the S cycle (Figure 4D).

Fungal OTUs were assigned to trophic groups and subsequently subdivided into specific ecological guilds using the FUNGuild database. Table 2 lists all the trophic modes identified in this study, and Supplementary Figure 6 shows the guilds that differed significantly among treatments. Saprotrophic fungi dominated in the SC and AC treatments, accounting for 24.13 and 27.03% of all fungi (Table 2). In contrast, pathotroph-saprotroph-symbiotroph fungi dominated in the AR treatment (30.91%; Table 2). The relative abundance of saprotroph-symbiotroph, pathotroph-saprotroph-symbiotroph, and pathogen-saprotroph-symbiotroph fungi were significantly higher in the AR site than in the other sites (Table 2). Regarding the ecological guilds, the relative abundance of 13 guilds such as ectomycorrhizal-undefined saprotroph, lichen parasite, animal pathogen-endophyte-lichen parasite-plant pathogen-soil saprotroph-wood saprotroph, endophyte-litter saprotroph-soil saprotroph-undefined saprotroph, and fungal parasite-undefined saprotroph significantly increased in AR, while endophyte-undefined saprotroph fungi were more abundant in SC and AC (Supplementary Figure 6). In addition, arbuscular mycorrhizal fungi (AM) and ectomycorrhizal fungi (EM) had the highest abundance in the SC and AR treatments, respectively.

**TABLE 2:** Differences in the relative abundance (%) of fungal functional trophic modes in tea nursery soils under different cropping types as inferred from the FUNGuild pipeline.

Trophic mode	SC	AC	AR
Symbiotroph	0.66 ± 0.32a	0.25 ± 0.05a	0.29 ± 0.17a
Saprotroph	24.13 ± 4.15a	27.03 ± 9.44a	19.88 ± 6.74a
Pathotroph	3.83 ± 2.60a	1.43 ± 0.38a	4.73 ± 2.15a
Saprotroph-Symbiotroph	7.32 ± 3.38a	4.94 ± 2.10a	14.27 ± 1.86b
Pathotroph-Symbiotroph	0.16 ± 0.05a	0.18 ± 0.08a	0.18 ± 0.07a
Pathotroph-Saprotroph	0.59 ± 0.62a	0.15 ± 0.06a	0.22 ± 0.28a
Pathotroph-Saprotroph- Symbiotroph	17.76 ± 3.18a	18.21 ± 2.19a	30.91 ± 1.85b
Pathogen-Saprotroph- Symbiotroph	0.00 ± 0.01a	0.00 ± 0.00a	0.86 ± 0.03b

Values are presented as means ± SDs, different letters indicate significant differences among the treatments (*P* < 0.05).

The RDA analysis showed that the three treatments formed distinct bacterial community function (Figure 3C). Fungal community functions under SC and AC treatments were similar and clearly differentiated from AR on the first axis (Figure 3D). The first and second axis jointly explained more than 82 and 63% of the total variation in the bacterial and fungal community function, respectively (Figures 3C,D). Soil Hp and Lp were the most crucial drivers of bacterial community function, and SOC and TN were closely linked with fungal community function (Supplementary Table 10).

### Relationships between soil physicochemical properties and microbes

**FIGURE 5:**
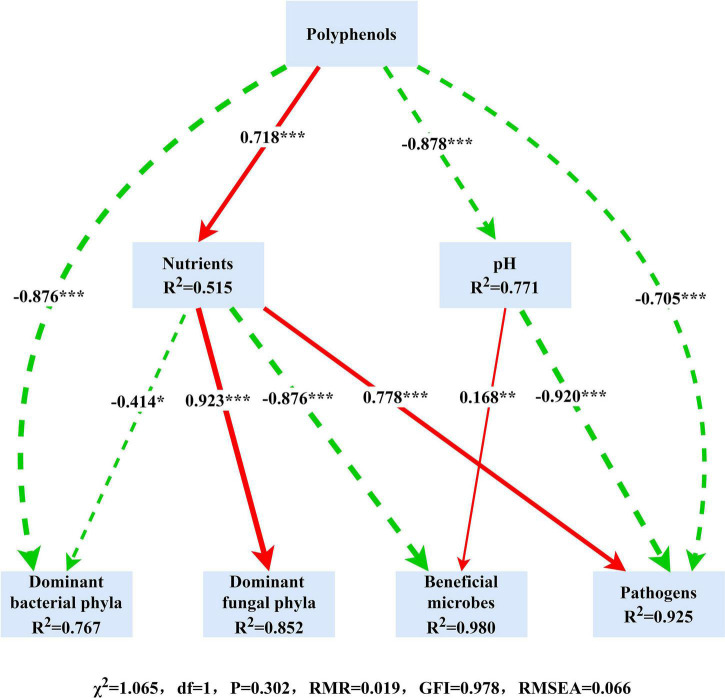
A structural equation model (SEM) assess the links between soil properties, microbial community, and the potential beneficial/pathogenic microbes. The red lines and green dashed lines indicate positive and negative links, respectively. The width of arrows indicates the strength of significant standardized path coefficients (*P < 0.05, **P < 0.01, ***P < 0.001).

**FIGURE 6:**
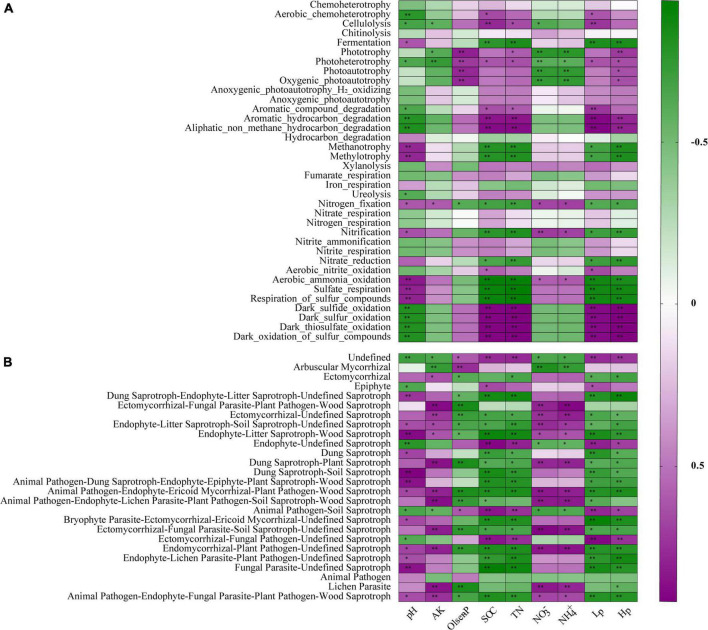
Correlations between the microbial functionalities and environmental variables based on spearman’s rank analysis. The functional groups related to C, N, and S cycles were selected for bacterial community **(A)**, and functional guilds with significant differences among treatments were selected for fungal community **(B)**. R in different colors to show, the right side of the legend is the color range of different *R* values. The values of *P* ≤ 0.05, *P* ≤ 0.01 are marked with “*,” and “**,” respectively. Abbreviations are as listed in Table 1.

It became evident that bacterial and fungal communities were largely influenced by the crop type, and we began to explore the role of the resulting variation in environmental factors in the composition and function of microbial communities. We used SEM to elucidate the role of soil polyphenols in predicting soil physicochemical properties, the relative abundance of dominant phyla, plant beneficial microbes, and pathogens. SEM result demonstrated that soil polyphenols (Hp and Lp) directly and indirectly affect bacterial and fungal community by altering soil nutrient availability and soil acidity (Figure 5). Soil polyphenols rather than soil nutrients or pH, had the strongest and direct effect on bacterial communities (−0.876; Figure 5). In addition, soil polyphenols also directly or indirectly interfere with the growth of plant beneficial (*Bacillus*, *Mortierella*, *Bradyrhizobium*, *Trichoderma*) and pathogenic microbes (*Fusarium*, *Pseudopestalotiopsis*). Spearman’s correlation analysis showed that, of 13 bacterial phyla, 52 genera and 13 fungal phyla, 26 genera presented significant correlations with edaphic properties (Supplementary Table 11 and Supplementary Figures 4, 5). Soil Hp (40 bacterial genera in total) associated with the greatest number of bacterial taxa, followed by TN (38 bacterial genera) and SOC (37 bacterial genera; Supplementary Figure 4). Besides, soil TN (15 fungal genera in total), followed by NH_4_^+^, NO_3_^–^ (14 fungal genera in each), were the edaphic properties with the highest number of soil fungal groups correlated with them (Supplementary Figure 5). SOC, TN, Lp, Hp, and pH were significantly correlated with bacterial functional groups engaged in the C, N, and S cycle (Figure 6A). In general, ectomycorrhizal fungi had positive correlations with pH, AK, NO_3_^–^, and NH_4_^+^, but negative relationships with Olsen-P, SOC, TN, Lp, and Hp. Saprotrophs were generally negatively related to SOC, TN, Hp, Lp, and Olsen-P but positively correlated with soil pH, AK, NO_3_^–^, and NH_4_^+^ (Figure 6B). Overall, different cropping types indirectly regulate soil nutrients and soil pH by modulating soil polyphenol dynamics, which subsequently affects soil microbial communities and community functions.

## Discussion

### High polyphenol promotes soil nutrients availability in continuously cropped tea nurseries

**FIGURE 7:**
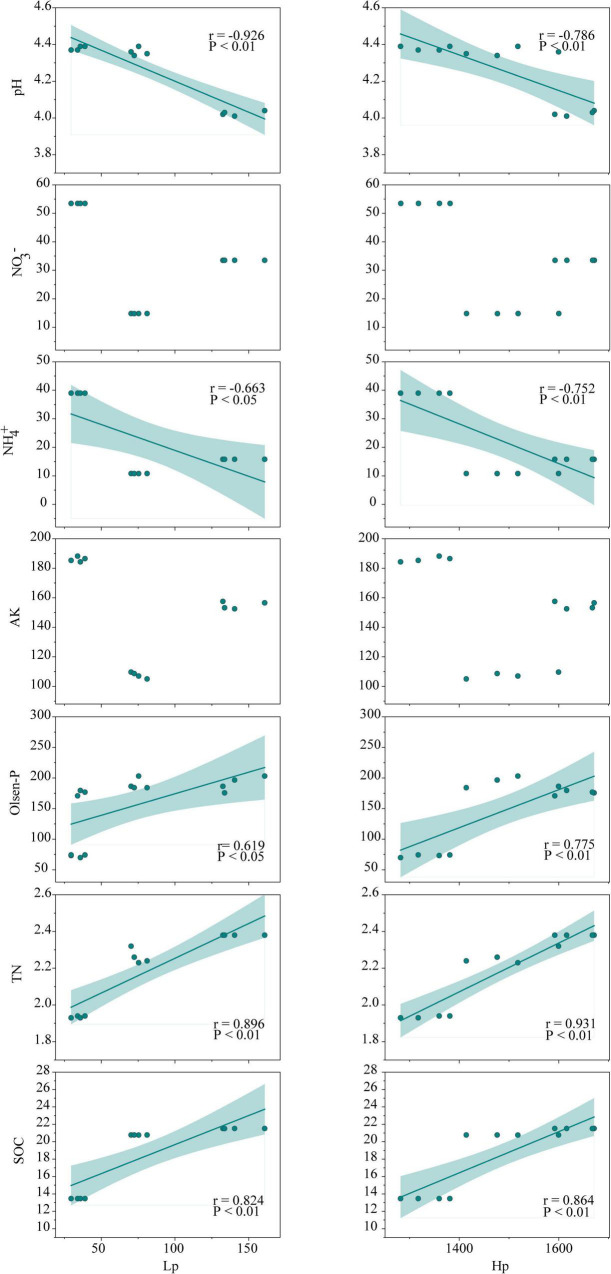
Relationships between soil polyphenols (Hp and Lp) and environmental variables (pH, TN, SOC, AK, Olsen-P, NO_3_^–^, NH_4_^+^) in the tea nursery soils. Green lines represent correlation (*P* < 0.05) across three planting patterns. Shaded areas represent the 95% confidence intervals for the regression lines. The abbreviations are as presented in Table 1.

Soil organic carbon is an important factor in characterizing soil quality and soil sustainability, and SOC reduction is frequently reported in successive monoculture systems, which is an important indication of soil degradation (Reeves, 1997; Alvarez et al., 1998). Interestingly, significant augmentations of SOC, TN, and Olsen-P were observed in continuously cropped tea nurseries in comparison to rotational ones (Table 1), which is distinct from the results observed in other ecosystems. From the results of our previous study in long-term monoculture tea plantations (Fan et al., 2016), we suggest that there is a strong connection between soil nutrient status and polyphenol dynamics in tea nurseries. First, the enhancement of SOC and TN in continuously cropped tea nurseries could benefit from the accumulation of soil Hp and Lp (Figure 7). This is mainly because, on the one hand, polyphenols can inhibit the decomposition of tea leaf litter (Fan et al., 2017), which is agreed with the results observed in polyphenol-rich pigmy forests and *Eucalyptus* plantations (Northup et al., 1998; Cizungu et al., 2014). In addition, polyphenols may also hinder soil organic matter decomposition by depressing soil mineralization or influencing the composition and activity of soil decomposers (Vitousek, 2000; Fierer et al., 2001; Kraus et al., 2003). On the other hand, polyphenols can be involved in abiotic humification through polyphenol polymerization and integrated polyphenol-Maillard reaction (Hardie et al., 2010; Zhang et al., 2021). The study of Sivapalan (1982) confirmed that the return of polyphenol-rich tea residues caused the formation of considerable amounts of N-rich humus. Furthermore, studies in pigmy forest and legume crop fields have also demonstrated that high polyphenol concentrations might help maintain P availability (Tsai and Phillips, 1991; Northup et al., 1998). In contrast to the increase of TN, soil NH_4_^+^, and NO_3_^–^ contents in continuous tea nursery soils were significantly reduced (Table 1). This is most likely due to the inhibitory effect of polyphenols on key processes of the N cycle including nitrification and N mineralization (Bradley et al., 2000; Tang et al., 2021), which was also verified by the results of functional changes in bacterial community (Figures 4E, 6A). In exception to soil nutrients, a significant negative correlation between polyphenols and soil pH was also observed in our research (Figure 7). Therefore, we suggest that polyphenol dynamic is a decisive factor in regulating soil physicochemical indicators in tea nurseries, which is also an important feature that distinguishes tea nurseries from other ecosystems.

### Crop rotation facilitates soil nutrient-cycling bacteria and forms a healthier microbial community in tea nurseries

The significant reduction of SOC, TN, and Olsen-P in rotational tea nursery soils drastically stimulated the growth of nutrient cycling-related bacteria, including *Verrucomicrobiota*, *Gemmatimonadota*, *Myxococcota*, *Desulfobacterota*, and *Methylomirabilota*, whose relative abundance was remarkably higher in AR than that in AC and SC (Supplementary Table 2). Some *Verrucomicrobiota* species can live in infertile soils through decomposing the recalcitrant carbon (Fierer et al., 2007; Trivedi et al., 2013), and some are involved in methane oxidation in acidic environments (Dunfield et al., 2007; Sharp et al., 2014). *Gemmatimonadota* are considered as SOM-miners, which may utilize the energy from the degradation of fresh organic matter or light fractions of SOM to mine old SOM for nitrogen (Razanamalala et al., 2018). Besides, members of *Methylomirabilota* and *Desulfobacterota* are engaged in soil N (Ettwig et al., 2010; Adrien et al., 2017) and S cycling (Pearson, 2003), respectively. The significant upregulation of these nutrient cycle-associated bacteria in AR implies that the soil microbial community responds rapidly to the reduction in soil available nutrients (Ren et al., 2020), reflecting the strong functional compensation by soil microbes to better support plant competitive and adaptive abilities (Yan et al., 2017; Ling et al., 2022). Unlike rotation, continuous cropping significantly increased the relative abundance of stress-resistant bacteria in the soil. Members of *WPS-2*, *AD3* (belonging to *Chloroflexi*), and *Ktedonobacteria* (belonging to *Chloroflexi*) can live in the oligotrophic soil and extreme environments (Jiang et al., 2016; Ji et al., 2017), and some *Acidimicrobiia* and WPS-2 favor acidic habitats (Qu et al., 2020). The increase of plant stress tolerant bacteria indirectly mirrors the continuous soil deterioration in successive mono-cropped tea nurseries.

The results of changes in the functional groups of soil bacteria under different crop types echoed the variations in bacterial community composition. Function groups related to the N cycling and S cycling increased significantly in AR compared with SC and AC (Figures 4B,C). The relative abundance of functional groups involved in C cycle was higher in SC and AC than that in AR, which was mainly due to the increase in SOC in the two continuously cropped tea nurseries, which directly provided a large amount of carbon source for microorganisms and promoting carbon assimilation and utilization by bacteria, thus leading to the increase of heterotrophic bacteria. Previous studies have suggested that crop rotation promotes the conversion of soil nutrients to better satisfy plant growth requirements compared with continuous cropping (Fauque and Ollivier, 2004; Richardson et al., 2009; Richard et al., 2017). This is generally consistent with the results found in this study, but the higher accumulation of available nutrients (especially SOC and TN) in the soil of continuously cropped tea nurseries compared to other continuous cropping systems may effectively mitigate the decline in carbon cycling function with bacterial involvement.

The changes in fungal community composition and function also revealed further improvement in soil function under crop rotation. First, phyla *Mortierellomycota* and *Mucoromycota* presented the highest relative abundance in AR, indicating a healthier habitat under crop rotation compared to the continuous cropping system (Zhang et al., 2011; Bonfante and Venice, 2020). Second, the growth of pathotroph-saprotroph-symbiotroph, saprotroph-symbiotroph, and pathogen-saprotroph-symbiotroph fungi was stimulated by crop rotation (Table 2), and the increment of these mixed trophic fungi revealed more diverse fungal functional profile, indicating that crop rotation is more advantageous for maintaining the stability of ecological functions over continuous cropping (Maherali and Klironomos, 2007). The lower SOC concentration in AR stimulated the growth of facultative saprotroph fungi because saprotrophs can decompose recalcitrant C into simple carbohydrates to cope with the harsh or infertile environments (Dighton, 2003). There is a significant increment in the relative abundance of functional guilds ectomycorrhizal, ectomycorrhizal-undefined saprotroph, and ectomycorrhizal-fungal parasite-soil saprotroph-undefined saprotroph fungi under crop rotation. Ectomycorrhizal fungi play an essential role in the nutrient and C cycle (Smith and Read, 2008), and they can mobilize nutrients from organic substrates for host growth (Talbot et al., 2013), mainly to improve the P nutrition for the host plants (Godbold, 2004); this is in line with the significant reduction in Olsen-P, SOC, and TN in the AR treatment. The proportions of endophytes (distributed in eight functional guilds, such as endophyte-litter saprotroph-soil saprotroph-undefined saprotroph, endophyte-lichen parasite-undefined saprotroph, animal pathogen-endophyte-lichen parasite-plant pathogen-soil saprotroph-wood saprotroph, endophyte-litter saprotroph-wood saprotroph) significantly increased in AR (Supplementary Figure 6). Generally, endophytic microorganisms can produce beneficial bioactive metabolites to assist plant growth in stressful environments (Srivastava et al., 2020), including pathogen depression (Sturz et al., 2000). No significant difference was found for the fungal functionality in the relative abundance of different trophic groups between the SC and AC treatments, indicating cutting season had little effect on fungal functions under continuous cropping system. This explained why the fungal functions from SC and AC soil samples were grouped together and away from AR (Figure 3D). We concluded that the community function of soil fungi in the rotation tea nursery was improved, and the growth of beneficial fungi such as EM fungi and endophytic fungi was promoted, thus making the crop rotation system more favorable to plant growth than the continuous cropping system.

### Rotational cropped tea seedlings recruited more potential beneficial microbes in soils

Soil bacteria and fungi play essential roles in affecting plant growth in response to diverse abiotic and biotic stresses. In this study, *Bacillus*, *Trichoderma*, *Saitozyma*, *Mortierella* and *Acremonium*, *Metarhizium* exhibited a higher relative abundance in AR than that in AC and SC (Supplementary Figure 7). *Bacillus* is typical bio-control bacteria that have been widely used as biofertilizers and biopesticides (Borriss, 2020). *Saitozyma* is yeast-like fungus that can participate in the mineralization of organic matter through fermentation or respiration, the transformation of nitrogen compounds and inorganic sulfur, and the enhancement of plant defense against pathogens (Botha, 2011; Prakash et al., 2018). *Trichoderma* species are vital in suppressing plant pathogen and facilitating plant growth in the agroecosystem (Vinale et al., 2008; Contreras-Cornejo et al., 2016). The dramatic increase in the relative abundance of *Trichoderma* in AR was mainly attributed to the increase of two species, *T. asperellum* and *T. virens*, both of which can protect plant from *Fusarium* wilt (Jogaiah et al., 2018; Hasan et al., 2020). In addition, endophytes of the genus *Acremonium* are resistant to herbivores and nematodes (West et al., 1988; Breen, 1994). Since *Mortierella* was reported to grow better in healthy soils than that containing *Fusarium* wilt (Xiong et al., 2017; Yuan et al., 2020). Therefore, the increase in the relative abundance of these potential beneficial microbes illustrated the notable improvement of the tea nursery soil under the rotational cropping system.

*Fusarium* is well-known pathogenic fungi that cause plant diseases in a lot of plants including strawberries and tea plants (Lastra et al., 2018; Cheng et al., 2020). Interestingly, the highest abundance of *Fusarium* was observed in AR treatment where tea root rot did not occur extensively. Further analysis revealed that the *Fusarium* observed in this study were mainly unclassified *Fusarium* spp. (SC: 8.25%, AC: 9.70%; AR: 15.36%) and *F. solani* (SC: 0.29%; AC: 0.36%; AR: 3.07%), which indicated that the augmentation of *Fusarium* in the rotational cropped tea nursery resulted from the increment of unclassified *Fusarium* spp. *Fusarium solani* is the main pathogen causing root rot in tea trees and strawberries, thus we speculate that the enhancement of *F. solani* in AR may be derived from residues after strawberry rotation (Lastra et al., 2018). Furthermore, the presence of *Trichoderma* in AR could reduce disease incidence by eliciting *Fusarium* resistance in tea trees (Reeslee, 2008; Jogaiah et al., 2018), which might explain why the highest abundance of *F. solani* did not cause suppression of the growth of tea seedlings in rotational tea nurseries. As for the surge in the relative abundance of unclassified *Fusarium* spp. under crop rotation, we presume that it may be due to the well-grown tea seedlings that facilitated its propagation. Furthermore, despite being a potential pathogen, *Fusarium* has various biological functions: some members are functioning in the N_2_O release and residue recycling in tea plantation soils (Cheng et al., 2020; Zheng et al., 2020); some are cellulose- (Yu, 2003) and polyphenols-decomposers (Lewis and Starkey, 1969); some *Fusarium* isolated from healthy tea plant even has broad-spectrum antimicrobial properties (Liu, 2019). Therefore, the function of unclassified *Fusarium* spp. in AR treatment can be clarified in future studies by methods such as isolation and culture to gain insight into *Fusarium*’s role played by Fusarium in the unique tea nurseries and tea plantation ecosystems. The relative abundance of pathogenic fungi *Pseudopestalotiopsis* and *Talaromyces* was higher in AC compared with SC and AR (Supplementary Figure 7). *Pseudopestalotiopsis* species found in this study were mainly *Pseudopestalotiopsis theae* that can cause tea leaf spot disease (Maharachchikumbura et al., 2016), while *Talaromyces* are pathogens in continuously monoculture *Radix Pseudostellariae* rhizosphere soils (Wu et al., 2016). The decrease in the relative abundance of potentially beneficial microbes and the increase in the relative abundance of pathogenic fungi, unbalancing the microbial community, which may be the primary reason for the extremely high mortality of tea seedlings in AC. However, as the interactions between soil microorganisms are complicate, the interactions between potential pathogenic and growth-promoting microbes in tea nursery soils and the mechanisms by which they regulate the growth of tea seedlings can be explored by using isolation and microbial co-culture in the future.

Additionally, we found higher levels of *Bradyrhizobium* and *Penicillium* in continuously cropped tea nursery soils than that in rotational ones (Supplementary Figure 7). Among them, *Bradyrhizobium* is root nodule bacteria with N_2_-fixing capacity (López et al., 2017) and its germination can be induced by root secreted flavonoids (Garg and Geetanjali, 2007). Given that *Penicillium* is one of the reported polyphenol-decomposers (Lewis and Starkey, 1969), then the enhancement of *Bradyrhizobium* and *Penicillium* in AC and SC is likely response of the microbial community to polyphenols accumulation under the continuous cropping system.

### Polyphenols are the pivotal factors in regulating soil microbial communities in tea nursery soils

Changes in cropping systems are considered to be the major disturbance to artificial ecosystems, which could profoundly alter edaphic properties and regulate microbial diversity, community composition, and function (Lyu et al., 2020; Zhao et al., 2020). In the current study, the α-diversity of the bacterial communities (OTU richness and Shannon index) in rotational tea seedling soils was significantly higher than in continuously grown tea seedling soils, probably because rotation directly increases plant diversity, leading to an increase in litter and root exudates species (Hooper et al., 2000; Eisenhauer, 2016), and the increase in substrate diversity contributes to an increase in soil microbial diversity (Venter et al., 2016). However, we found no significant differences in the α-diversity of fungal communities among different crop types, which is consistent with the results observed in long-term consecutive monoculture soybean soils (Liu et al., 2020). It has been noted that fungi are not sensitive to the environmental changes caused by different cropping systems compared with bacteria (Venter et al., 2016), as confirmed by the weak correlation between fungal α-diversity indices and soil properties in this study, this may explain why the alpha diversity of fungi did not show significant differences among the three treatments. As this study is a relatively short-term field trial, the results of long-term trials are needed to complement the results regarding the response of microbial diversity to different cropping systems.

The driving role of environmental factors on microbial compositions has been emphasized in tremendous research (Pennanen et al., 1999; Rousk et al., 2010), as in this study, changes in soil physicochemical properties resulting from cropping pattern changes also played an essential role in shifting microbial community composition and function. Differing from many previous findings that available soil nutrients or pH are the most effective factors governing microbial community assembly at the regional scale (Castrillo et al., 2017; Ren et al., 2020), our study revealed polyphenols are the dominant factor regulating soil microbial community in tea nursery soils. As an important secondary metabolite, polyphenols can directly regulate microbial growth and indirectly influence microbial community assembly by affecting soil nutrients, pH and other physicochemical variables in polyphenol-rich tea nurseries (Figure 5). Most of the potential beneficial and pathogenic microbes found in tea nursery soils were closely associated with soil polyphenols (Supplementary Figures 3, 4). For instance, the relative abundance of *Bacillus*, *Acremonium*, and *Saitozyma* were significantly and negatively correlated with Hp and Lp concentrations, so the reduced polyphenols in AR favored the proliferation of these plant growth-promoting bacteria and fungi. *Fusarium* displayed significant negative correlations with Lp and Olsen-P and positive correlations with AK, NO_3_^–^, and NH_4_^+^, suggesting that the low-molecular-weight polyphenols may inhibit *Fusarium* colonization, while inorganic nitrogen could promote its growth, which explains the significant increase in the relative abundance of *Fusarium* in AR. In addition, the intense linkages between polyphenols and microbial community functions also indicate that polyphenols have great ecological importance in soil nutrient cycling, plant growth and stress resistance in tea nurseries (Figures 6A,B). Similar results were observed in long-term monoculture tea plantations (Arafat et al., 2020), cucumber fields (Zhou et al., 2012), and arctic-alpine ecosystems (Baptist et al., 2010). Therefore, it is important to explore the ecological role of polyphenols in tea plantation ecosystems in-depth for sustainable production in tea plantations. However, the reasons for the accumulation of large amounts of polyphenols in tea plantations are poorly understood, and the detailed mechanisms by which tea polyphenols affect microbial community assembly are not well-defined. In the future, by studying the tea plant- polyphenols-microbes interaction by dilution culture method and rhizosphere microbial reorganization experiments, we can broaden the knowledge of the ecological roles of tea polyphenols in the tea plantation ecosystem.

## Conclusion

In this study, we found that the dynamics of soil polyphenols under different crop types affected soil nutrient status and thereby shifted soil microbial diversity, composition and functions. The augmentation of the relative abundance of beneficial soil microbes and the reduction of pathogens in AR reveal a healthier microbial community under crop rotation. Comparatively, the increment of acidophilic bacteria and stress-resistance microbes in the AC and SC treatments illustrated that the microbial community assembly pattern in the continuously cropped tea nursery soils aimed to improve plant resistance. Furthermore, the relatively lower nutrient status in the AR treatment facilitated the nutrient cycling-related functions of bacteria and increased the growth of mixed-trophic fungi, endophytic fungi and EM fungi, which further satisfied the nutrient requirements of tea seedlings. Therefore, we suggest that the primary mechanism of crop rotation to alleviate soil succession disorders in tea nurseries is to reduce the accumulation of soil polyphenols to regulate soil biotic and abiotic processes, thus facilitating tea seedlings’ growth. Our research reveals the strong associations between microbial communities and plant growth, highlighting the ecological role of secondary metabolites in the terrestrial ecosystem. In future experiments, we need to integrate the above-ground and below-ground to investigate plant-polyphenols-microorganisms interactions in depth to assess the relative contribution of secondary metabolites in soil and plant health.

## Data availability statement

The datasets presented in this study can be found in online repositories. The names of the repository/repositories and accession number(s) can be found below: https://www.ncbi.nlm.nih.gov/, PRJNA738594.

## Author contributions

DF designed the experiments, did the statistical and microbial community analysis, and wrote the manuscript. ZZ performed the soil physicochemical analysis. YW and JM selected the experimental site and collected all the samples. XW revised the manuscript and obtained funding. All authors contributed to the article and approved the submitted version.

## Footnotes

1
https://www.mothur.org/wiki/Download_mothur
