# Supplementary material for: Crop-type-driven changes in polyphenols regulate soil nutrient availability and soil microbiota
Source: Front Microbiol. 2022 Aug 24;13:964039. doi: 10.3389/fmicb.2022.964039 (PMC9449698; doi:10.3389/fmicb.2022.964039)
Supplement: Supplementary file 1 [file Data_Sheet_1.docx]

Supplementary Material

# Supplementary Figures and Tables

## Supplementary Figures


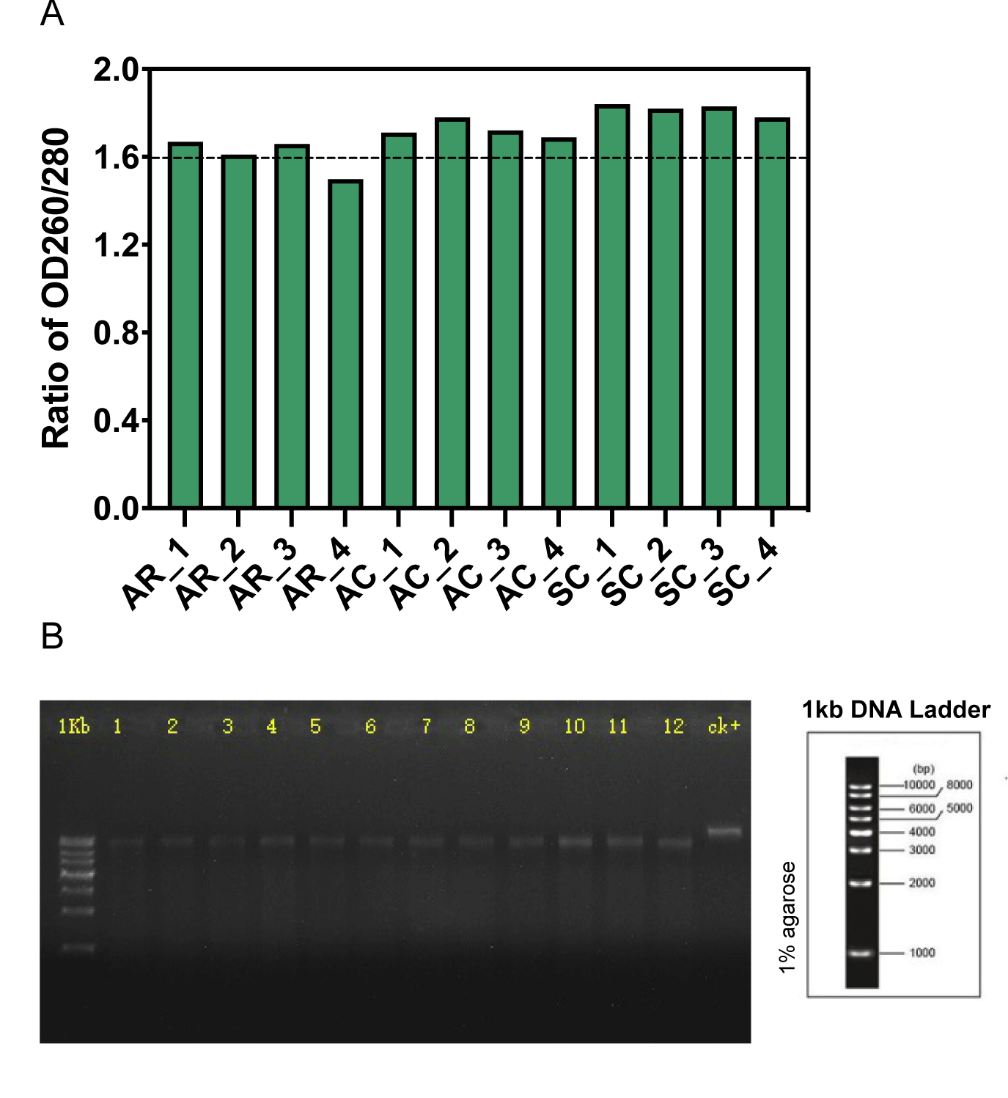


**Supplementary Figure 1.** The OD260/280 ratios (A) and the electrophoretogram (B) of the extracted soil DNA in tea nurseries under different cropping types. Electrophoresis was performed at 5V/cm voltage for 20 minutes. The numbers 1-12 in figure B correspond to AR_1 to SC_4 in figure A, respectively.


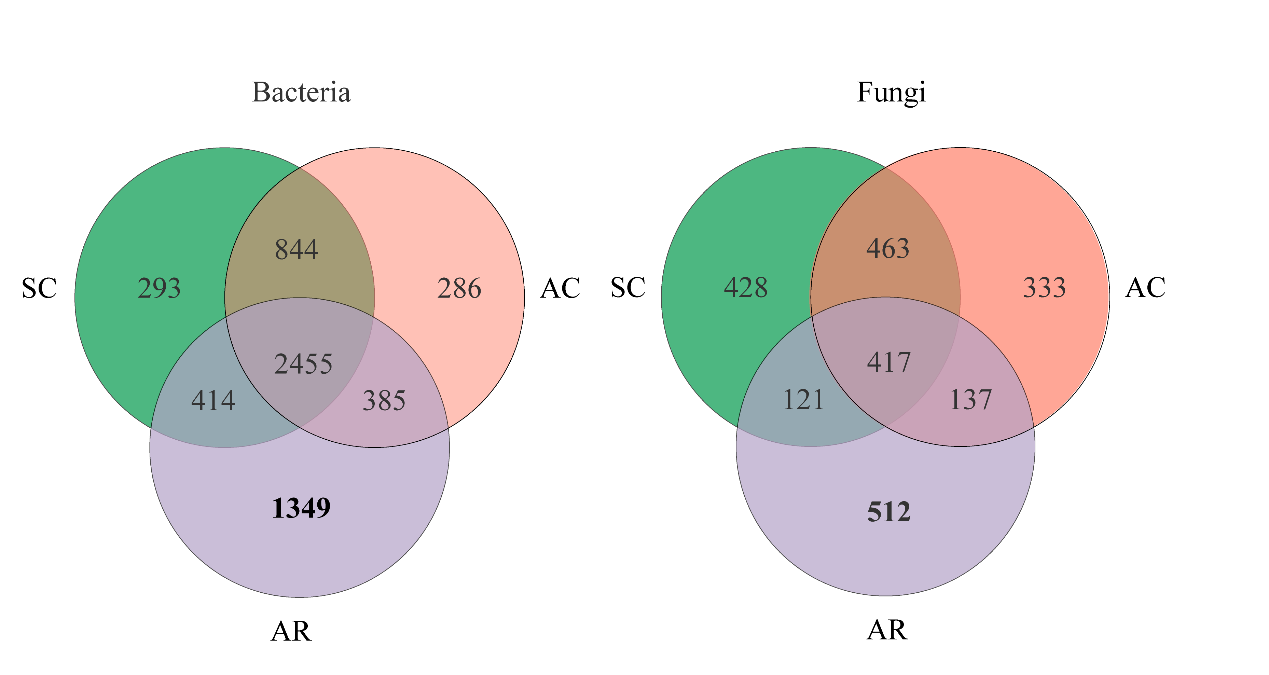


**Supplementary Figure 2.** Venn diagram showed the unique and shared soil bacterial and fungal OTUs from the continuously cropped tea nursery cutting in summer (SC) and autumn (AC), as well as rotational cropped tea nursery cutting in autumn (AR).


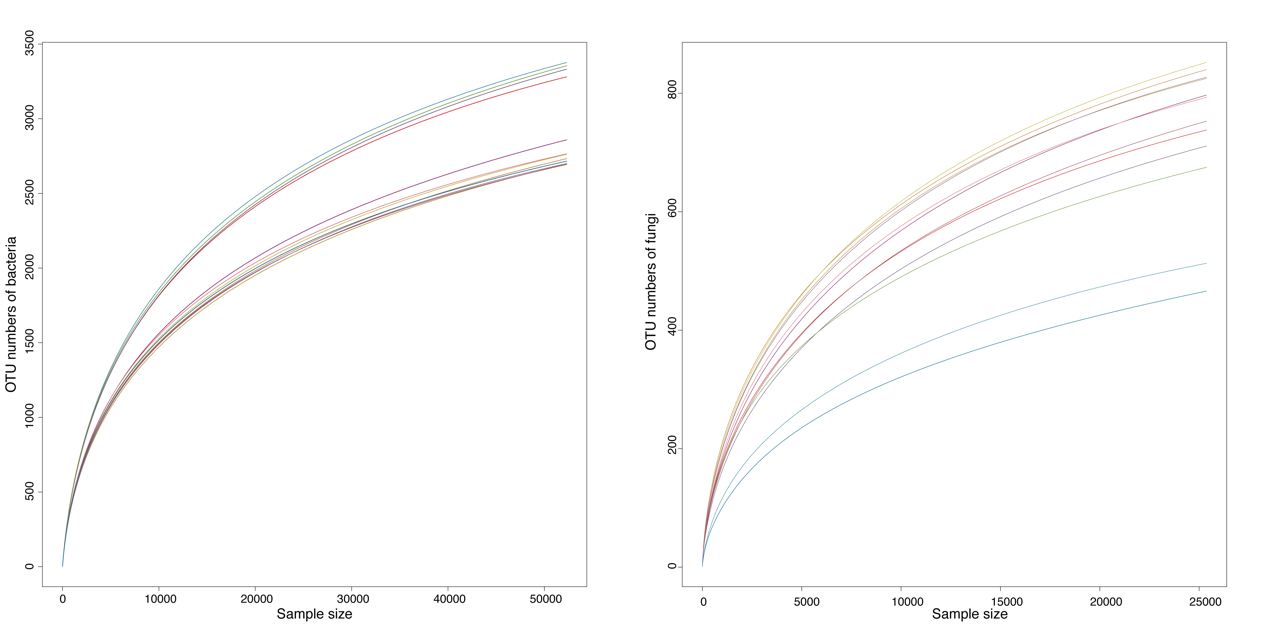


**Supplementary Figure 3**. The rarefaction curves of all sequences for bacteria and fungi under different cropping types.


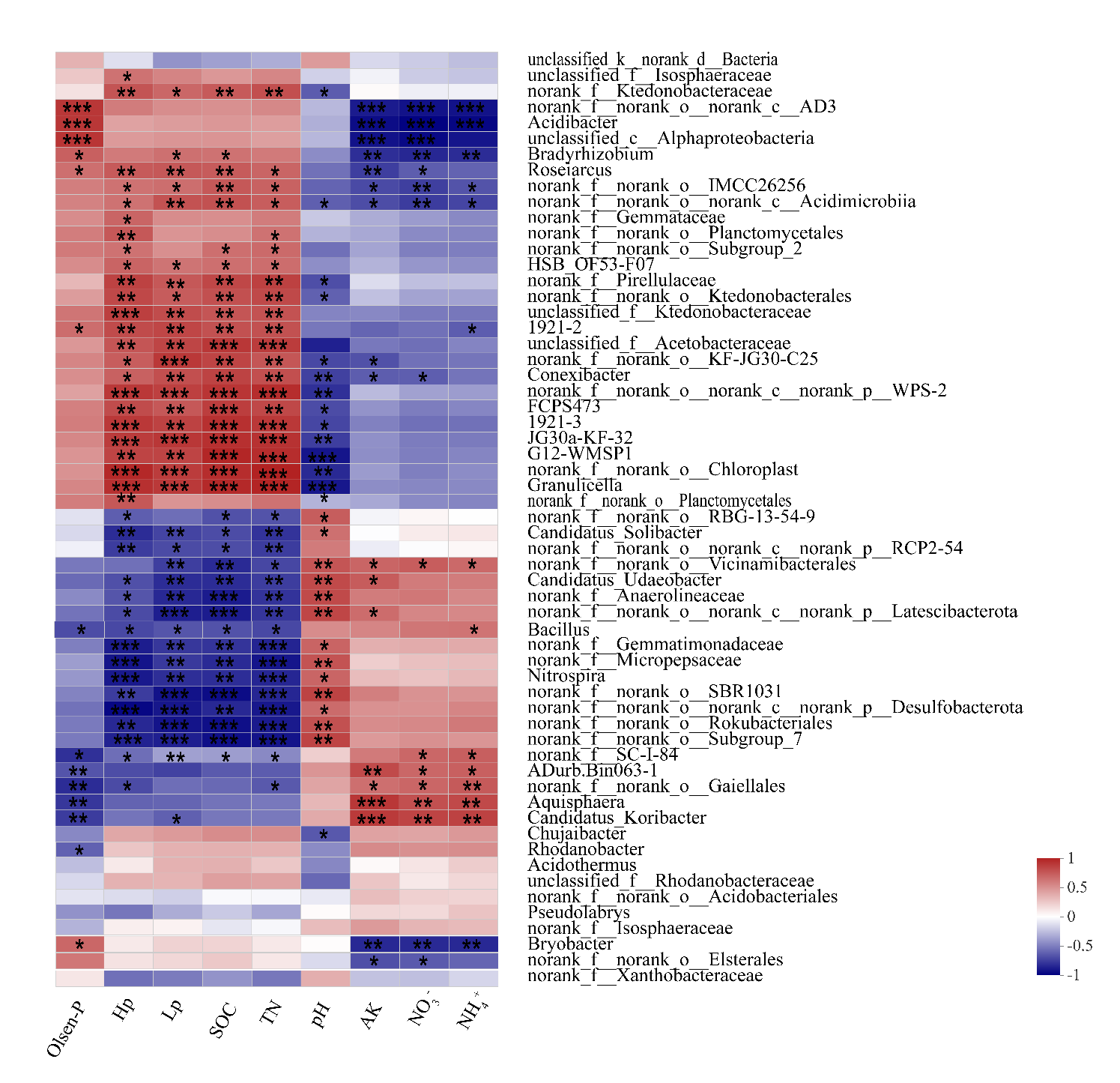


**Supplementary Figure 4.** Correlation heat map of the abundant bacterial genera (relative abundance>0.5%) and soil properties based on spearman’s rank analysis. R in different colors to show, the right side of the legend is the color range of different R values. The values of P≤0.05，P ≤ 0.01, and P ≤0.001 are marked with “*”, “**”, and “***”, respectively.


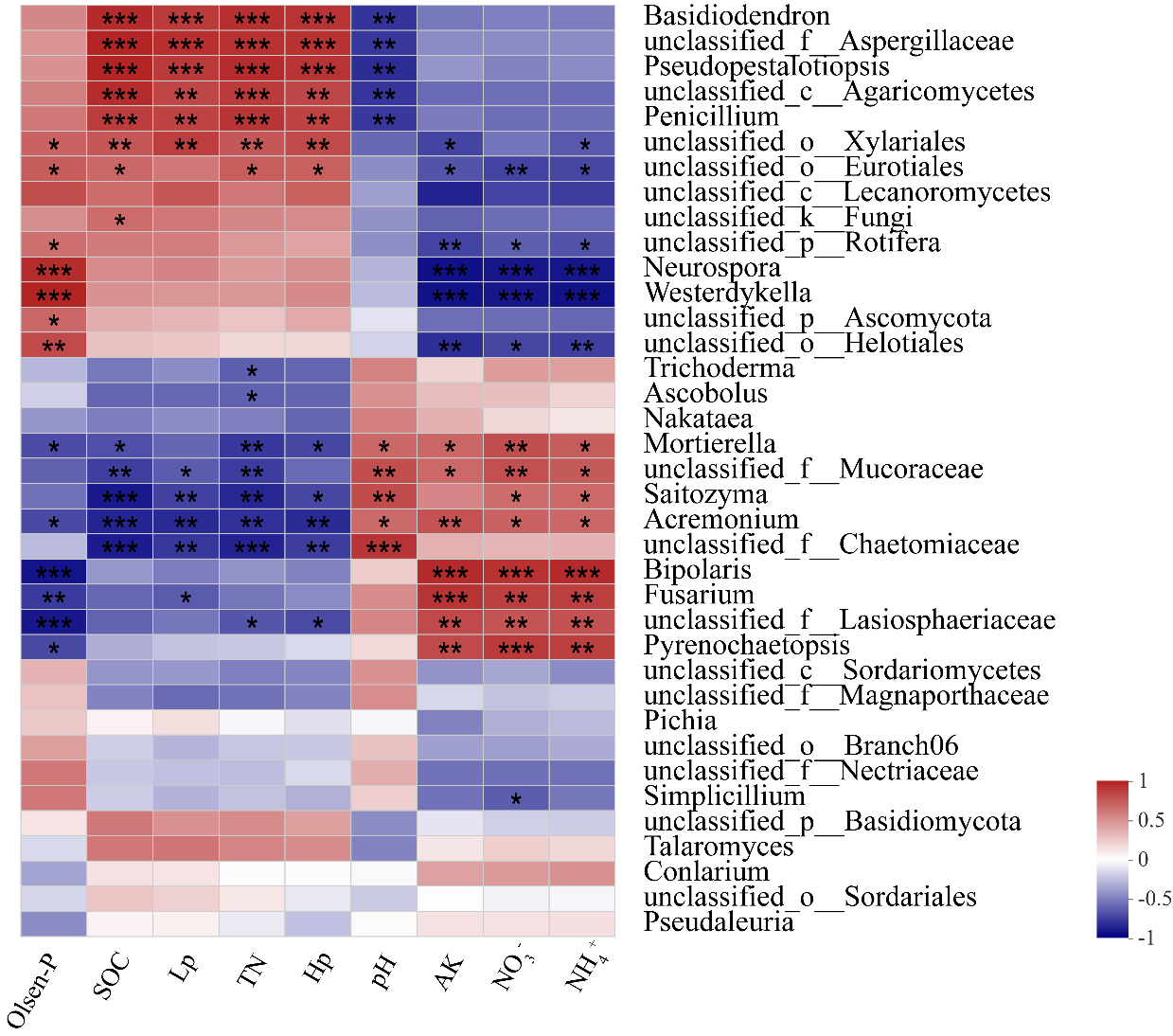


**Supplementary Figure 5.** Correlation heat map of the abundant fungal genera (relative abundance>0.5%) and soil properties based on spearman’s rank analysis. R in different colors to show, the right side of the legend is the color range of different R values. The values of P≤0.05，P ≤ 0.01, and P ≤0.001 are marked with “*”, “**”, and “***”, respectively.


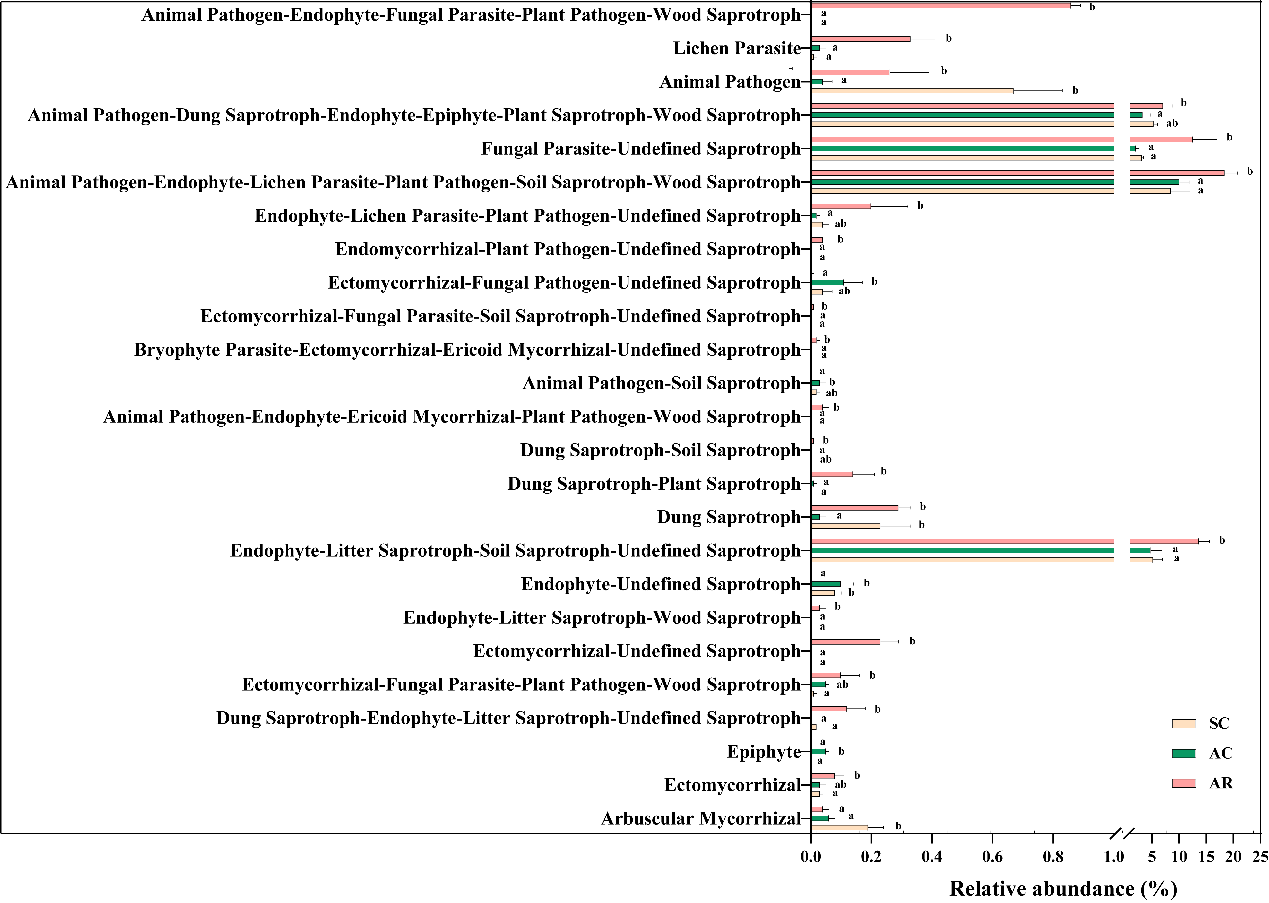


**Supplementary Figure 6**. Significant variation of fungal ecological guilds in tea nursery soils under different cropping patterns inferred by FUNGuild pipeline. Different letters represent the significant differences (P <0.05).


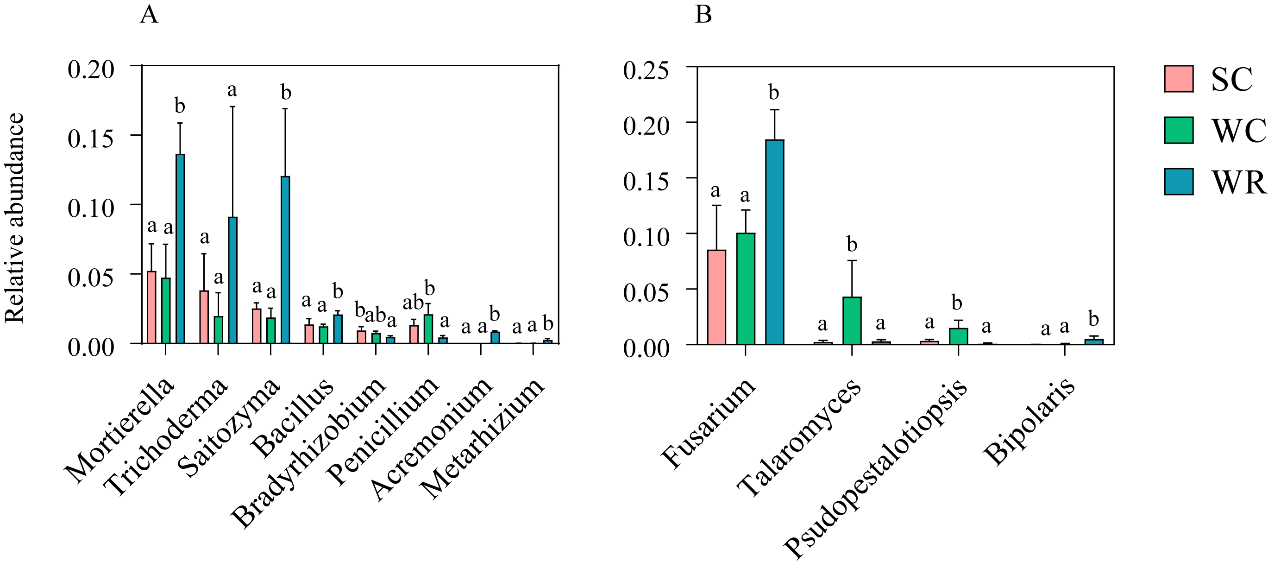


**Supplementary Figure 7**. The relative abundance of potential plant growth promoting microbes (A) and pathogens (B) in tea nurseries under different cropping types. Different letters represent the significant differences (P <0.05).

## Supplementary Tables

**Supplementary Table 1.** Spearman’s rank correlation coefficients and statistical significance between soil properties and microbial α-diversities.

| Soil properties | Bacteria | | Fungi | |
| --- | --- | --- | --- | --- |
|  | OTU richness | Shannon | OTU richness | Shannon |
| pH | **0.607*** | 0.328 | -0.261 | -0.127 |
| Lp | **-0.664*** | **-0.622*** | 0.545 | 0.399 |
| Hp | -0.524 | **-0.748**** | 0.469 | 0.245 |
| AK | **0.818**** | **0.944**** | -0.573 | -0.510 |
| Olsen-P | **-0.748*** | **-0.888**** | 0.545 | 0.413 |
| SOC | **-0.636*** | -0.462 | 0.520 | 0.350 |
| TN | **-0.600*** | -0.457 | 0.414 | 0.225 |
| NO_3_^-^ | **0.747**** | **0.894**** | -0.506 | -0.363 |
| NH_4_^+^ | **0.797**** | **0.940**** | -0.564 | -0.414 |

R values marked with “*” and “**” represent the P≤0.05 and P≤ 0.01, respectively.

**Supplementary Table 2.** Mean relative abundance of main bacterial phyla (relative abundance >1% at least in one sample) in the tea nursery soils under different planting patterns.

| Phylum | SC | AC | AR |
| --- | --- | --- | --- |
| Proteobacteria | 23.20±7.45a | 23.90±2.88a | 19.87±3.15a |
| Chloroflexi | 19.74±3.04b | 19.13±1.35b | 10.93±1.41a |
| Acidobacteriota | 14.17±2.69a | 14.71±4.29a | 16.18±1.75a |
| Planctomycetota | 7.31±3.29a | 8.44±0.64a | 8.74±2.02a |
| Actinobacteriota | 6.24±2.09a | 6.53±0.60a | 6.57±0.64a |
| Firmicutes | 2.48±0.73a | 2.38±0.27a | 3.26±0.41a |
| Gemmatimonadota | 1.67±0.51ab | 1.24±0.12a | 2.26±0.29b |
| Verrucomicrobiota | 1.80±0.53a | 1.79±0.29a | 6.09±1.99b |
| WPS-2 | 1.68±0.59b | 2.90±0.16c | 0.88±0.18a |
| RCP2-54 | 1.19±0.39a | 0.75±0.07a | 1.14±0.28a |
| Patescibacteria | 0.89±0.34a | 1.07±0.26a | 0.98±0.15a |
| Myxococcota | 0.88±0.28a | 0.58±0.08a | 1.50±0.20b |
| Cyanobacteria | 0.51±0.18a | 1.60±0.66b | 0.21±0.07a |
| Desulfobacterota | 0.19±0.03a | 0.06±0.01a | 1.17±0.15b |
| Methylomirabilota | 0.08±0.00a | 0.05±0.01a | 1.42±0.10b |
| unclassified_Bacteria | 15.34±3.71a | 12.49±1.49a | 14.14±1.70a |

Means are presented with standard deviation (*n* = 4). Different letters indicate significant differences at the P<0.05 level.

**Supplementary Table 3.** Mean relative abundance of main bacterial classes (relative abundance>1% at least in one sample) in the tea nursery soils under different cropping types.

| Class | SC | AC | AR |
| --- | --- | --- | --- |
| Gammaproteobacteria | 11.80±3.66a | 13.89±1.68a | 10.09±1.76a |
| Acidobacteriae | 13.64±2.67a | 14.34±4.30a | 12.19±1.14a |
| Alphaproteobacteria | 11.39±3.83a | 9.99±1.20a | 9.78±1.46a |
| Ktedonobacteria | 8.48±3.08b | 12.20±1.52b | 4.37±0.62a |
| AD3 | 7.83±0.76c | 4.23±0.47b | 1.83±0.43a |
| Planctomycetes | 6.86±3.10a | 7.91±0.65a | 8.01±1.87a |
| Thermoleophilia | 2.23±0.74a | 2.13±0.18a | 3.38±0.37b |
| Actinobacteria | 2.17±0.72a | 2.72±0.41a | 2.30±0.23a |
| Bacilli | 2.11±0.64a | 1.98±0.25a | 2.69±0.36a |
| Acidimicrobiia | 1.80±0.65b | 1.64±0.13b | 0.75±0.07a |
| Gemmatimonadetes | 1.61±0.5ab | 1.20±0.12a | 2.16±0.29b |
| Anaerolineae | 2.38±0.32b | 1.48±0.21a | 3.36±0.50c |
| RCP2-54_norank | 1.19±0.39a | 0.75±0.07a | 1.14±0.28a |
| Verrucomicrobiae | 1.54±0.50a | 1.35±0.23a | 5.58±1.72b |
| WPS-2_norank | 1.68±0.59b | 2.90±0.16c | 0.88±0.18a |
| Holophagae | 0.22±0.04b | 0.13±0.02a | 1.57±0.06c |
| Cyanobacteriia | 0.27±0.11a | 1.44±0.65b | 0.13±0.05a |
| Methylomirabilia | 0.08±0.00a | 0.05±0.01a | 1.42±0.10b |
| Vicinamibacteria | 0.21±0.12a | 0.19±0.02a | 1.52±0.55b |
| unclassified_Bacteria | 15.34±3.71a | 12.49±1.49a | 14.14±1.70a |

Means are presented with standard deviation (*n* = 4). Different letters indicate significant differences at the P<0.05 level.

**Supplementary Table 4.** Mean relative abundance of main bacterial genera (relative abundance > 0.5% at least in one sample) in the tea nursery soils under different cropping types.

| Genus | SC | AC | AR |
| --- | --- | --- | --- |
| unclassified_Bacteria | 15.34±3.71a | 12.49±1.49a | 14.14±1.70a |
| Chloroflexi_AD3_norank | 7.83±0.76c | 4.23±0.47b | 1.83±0.43a |
| Proteobacteria_KF-JG30-C25_norank | 4.22±1.73b | 3.77±0.19b | 1.23±0.33a |
| Proteobacteria_Elsterales_norank | 3.21±1.00a | 2.39±0.34a | 2.28±0.39a |
| Acidibacter | 3.59±0.71c | 2.52±0.20b | 1.38±0.31a |
| Acidobacteriota_Subgroup_2_norank | 4.81±1.68a | 4.91±1.71a | 2.73±0.56a |
| Bryobactermetha | 1.69±0.59b | 1.03±0.10ab | 0.89±0.21a |
| Acidobacteriota_Acidobacteriales_norank | 4.29±1.48a | 4.99±1.95a | 5.09±0.79a |
| Candidatus_Solibacter | 1.45±0.57a | 0.80±0.08a | 1.43±0.25a |
| Bacillus | 1.38±0.41a | 1.23±0.16a | 2.09±0.27b |
| RCP2-54_norank | 1.19±0.39a | 0.75±0.07a | 1.14±0.28a |
| Actinobacteriota_Gaiellales_norank | 1.11±0.41a | 1.09±0.14a | 1.90±0.21b |
| Chlorolexi_RBG-13-54-9_norank | 1.18±0.24b | 0.57±0.08a | 1.14±0.11b |
| Gemmatimonadota _Gemmatimonadaceae_norank | 1.04±0.32ab | 0.71±0.08a | 1.39±0.26b |
| Chloroflexi_JG30-KF-AS9_norank | 2.14±0.68ab | 2.81±0.36b | 1.69±0.49a |
| Proteobacteria_Xanthobacteraceae_norank | 0.89±0.33a | 0.62±0.07a | 0.70±0.09a |
| Acidothermus | 0.95±0.29a | 1.13±0.13a | 0.94±0.12a |
| Bradyrhizobium | 0.94±0.26b | 0.76±0.13ab | 0.47±0.10a |
| Proteobacteria_Micropepsaceae_norank | 0.85±0.26a | 0.55±0.07a | 0.97±0.26a |
| Proteobacteria_Alphaproteobacteria_norank | 0.81±0.27b | 0.54±0.07ab | 0.41±0.06a |
| Actinobacteriota_IMCC26256_norank | 0.79±0.30a | 0.68±0.07a | 0.49±0.05a |
| Roseiarcus | 0.74±0.30b | 0.63±0.08ab | 0.35±0.08a |
| Chujaibacter | 0.62±0.29a | 2.74±0.50b | 1.35±0.48a |
| Actinobacteriota_Acidimicrobiia_norank | 0.68±0.23b | 0.61±0.05b | 0.14±0.02a |
| Rhodanobacter | 0.59±0.24a | 1.76±0.32b | 1.37±0.24b |
| Planctomycetota_Isosphaeraceae_norank | 1.88±1.12a | 1.99±0.21a | 2.20±0.64a |
| Conexibacter | 0.58±0.18b | 0.53±0.01ab | 0.36±0.02a |
| WPS-2_norank | 1.68±0.59b | 2.90±0.16c | 0.88±0.18a |
| unclassified_f_Acetobacteraceae | 0.49±0.21ab | 1.01±0.60b | 0.21±0.05a |
| Pseudolabrys | 0.50±0.20a | 0.44±0.04a | 0.54±0.12a |
| HSB_OF53-F07 | 1.77±0.76ab | 2.17±0.19b | 1.14±0.29a |
| Proteobacteria_SC-I-84_norank | 0.38±0.22a | 0.57±0.09ab | 0.75±0.13b |
| Nitrospira | 0.45±0.17ab | 0.22±0.03a | 0.56±0.13b |
| Aquisphaera | 1.14±0.39a | 1.51±0.23a | 2.76±0.30b |
| Planctomycetota _Gemmataceae_norank | 1.21±0.46a | 1.24±0.05a | 0.85±0.26a |
| unclassified_f_Isosphaeraceae | 1.00±0.57a | 1.11±0.12a | 0.74±0.27a |
| unclassified_f_Rhodanobacteraceae | 0.39±0.23a | 0.66±0.09a | 0.58±0.05a |
| 1921-3 | 0.65±0.19b | 0.98±0.30b | 0.12±0.03a |
| unclassified_f_Ktedonobacteraceae | 1.03±0.56b | 1.30±0.14b | 0.30±0.08a |
| Planctomycetota_Pirellulaceae_norank | 0.53±0.20ab | 0.87±0.09b | 0.37±0.06a |
| JG30a-KF-32 | 0.56±0.25b | 0.91±0.12c | 0.11±0.01a |
| 1921-2 | 0.53±0.30b | 0.75±0.04b | 0.00±0.00a |
| Acidobacteriota_Subgroup_7_norank | 0.15±0.03a | 0.09±0.02a | 1.40±0.06b |
| FCPS473 | 0.41±0.15ab | 0.68±0.18b | 0.14±0.05a |
| Cyanobacteria_Chloroplast_norank | 0.25±0.10a | 1.43±0.66b | 0.10±0.05a |
| Planctomycetota_Planctomycetales_norank | 0.51±0.36a | 0.41±0.03a | 0.27±0.08a |
| Chloroflexi_Ktedonobacterales_norank | 0.38±0.22a | 0.66±0.03b | 0.18±0.10a |
| Chloroflexi_Anaerolineaceae_norank | 0.25±0.11a | 0.18±0.04a | 0.75±0.17b |
| ADurb.Bin063-1 | 0.24±0.11a | 0.28±0.14a | 0.61±0.05b |
| G12-WMSP1 | 0.27±0.12b | 0.54±0.14c | 0.05±0.02a |
| Chloroflexi_Ktedonobacteraceae_norank | 0.21±0.08a | 0.71±0.33b | 0.22±0.04a |
| Granulicella | 0.11±0.05a | 0.54±0.19b | 0.03±0.02a |
| Acidobacteriota_Vicinamibacterales_norank | 0.20±0.11a | 0.16±0.01a | 1.04±0.43b |
| Candidatus_Koribacter | 0.12±0.04a | 0.18±0.06a | 0.64±0.15b |
| Methylomirabilota_Rokubacteriales_norank | 0.04±0.01a | 0.02±0.01a | 0.97±0.09b |
| Desulfobacterota_norank | 0.04±0.01a | 0.02±0.01a | 0.59±0.11b |
| Chloroflexi_SBR1031_norank | 0.11±0.05a | 0.04±0.03a | 0.55±0.14b |
| Candidatus_Udaeobacter | 0.08±0.03a | 0.06±0.01a | 4.15±1.59b |
| Latescibacterota_norank | 0.01±0.01a | 0.00±0.00a | 0.57±0.19b |

Means are presented with standard deviation (*n* = 4). Different letters indicate significant differences at the P<0.05 level.

**Supplementary Table 5.** Mean relative abundance of main fungal phyla (relative abundance > 1% at least in one sample) in the tea nursery soils under different cropping types.

| Phylum | SC | AC | AR |
| --- | --- | --- | --- |
| Ascomycota | 65.32±6.34a | 62.14±17.28a | 59.18±0.44a |
| Basidiomycota | 12.33±5.82a | 17.31±7.97a | 16.01±4.34a |
| unclassified_Fungi | 15.54±1.77a | 14.21±7.04a | 7.93±2.99a |
| Mortierellomycota | 5.24±1.96a | 4.87±2.23a | 13.70±2.22b |
| Mucoromycota | 0.06±0.04a | 0.15±0.10a | 2.45±0.83b |

Means are presented with standard deviation (*n* = 4). Different letters indicate significant differences at the P<0.05 level.

**Supplementary Table 6.** Mean relative abundance of main fungal classes (relative abundance >1% at least in one sample) in the tea nursery soils under different cropping types.

| Class | SC | AC | AR |
| --- | --- | --- | --- |
| Sordariomycetes | 45.81±9.31a | 39.79±16.76a | 48.68±4.30a |
| unclassified_Fungi | 15.54±1.77a | 14.21±7.04a | 7.93±2.99a |
| Agaricomycetes | 6.46±5.68a | 10.98±7.19a | 1.02±0.20a |
| Mortierellomycetes | 5.24±1.954a | 4.78±2.38a | 13.69±2.22b |
| Eurotiomycetes | 4.98±3.65ab | 14.27±9.11b | 0.95±0.25a |
| unclassified_p_Ascomycota | 3.36±0.57a | 2.58±1.30a | 1.91±0.80a |
| Dothideomycetes | 3.27±1.26a | 4.13±3.69a | 4.07±1.34a |
| Tremellomycetes | 3.20±0.30a | 2.09±0.61a | 12.63±5.06b |
| unclassified_p_Basidiomycota | 2.49±0.39a | 3.83±1.78a | 2.02±0.87a |
| Pezizomycetes | 2.12±1.53b | 0.19±0.11a | 2.71±3.23b |
| Leotiomycetes | 2.03±0.80b | 0.97±0.48ab | 0.76±0.31a |
| Saccharomycetes | 1.96±3.89a | 0.01±0.01a | 0.02±0.01a |
| Lecanoromycetes | 1.70±3.03a | 0.17±0.11a | 0.00±0.00a |
| Mucoromycetes | 0.01±0.00a | 0.09±0.06a | 2.45±0.82b |

Means are presented with standard deviation (*n* = 4). Different letters indicate significant differences at the P<0.05 level.

**Supplementary Table 7.**  Mean relative abundance of main fungal genera (relative abundance > 0.5% at least in one sample) in the tea nursery soils under different cropping types.

| Genus | SC | AC | AR |
| --- | --- | --- | --- |
| unclassified_c_Sordariomycetes | 11.54±14.28a | 1.14±0.51a | 2.88±2.07a |
| Fusarium | 8.54±3.99a | 10.07±2.05a | 18.46±2.67b |
| unclassified_f_Chaetomiaceae | 5.29±0.72ab | 3.19±1.51a | 7.05±1.91b |
| Mortierella | 5.23±1.95a | 4.74±2.41a | 13.66±2.21b |
| Neurospora | 4.55±0.83c | 2.27±1.53b | 0.17±0.13a |
| Trichoderma | 3.82±2.64a | 1.97±1.69a | 9.13±7.93b |
| unclassified_c_Agaricomycetes | 3.73±3.49a | 6.36±4.10a | 0.29±0.11a |
| unclassified_p_Ascomycota | 3.36±0.57a | 2.58±1.30a | 1.91±0.80a |
| unclassified_o_Branch06 | 2.92±1.2a | 1.14±0.85a | 1.55±0.68a |
| unclassified_o_Eurotiales | 2.80±3.63a | 1.74±1.70a | 0.01±0.01a |
| Saitozyma | 2.53±0.39a | 1.87±0.66a | 12.06±4.85b |
| unclassified_f_Magnaporthaceae | 2.52±2.73a | 0.38±0.20a | 1.73±1.77a |
| unclassified_p_Basidiomycota | 2.49±0.39a | 3.83±1.78a | 2.02±0.87a |
| Basidiodendron | 2.15±2.24a | 4.05±2.98a | 0.00±0.00a |
| Pichia | 1.95±3.89a | 0.00±0.00a | 0.00±0.00a |
| Westerdykella | 1.88±0.30c | 0.51±0.25b | 0.05±0.02a |
| unclassified_c_Lecanoromycetes | 1.70±3.03a | 0.17±0.11a | 0.00±0.00a |
| unclassified_f_Nectriaceae | 1.52±0.85b | 0.30±0.13a | 0.46±0.16a |
| Penicillium | 1.33±0.41ab | 2.11±0.77b | 0.44±0.14a |
| unclassified_o_Sordariales | 1.27±1.10a | 2.00±1.65a | 1.25±0.93a |
| Ascobolus | 1.24±1.70a | 0.03±0.04a | 1.32±1.13a |
| unclassified_o_Helotiales | 0.99±0.35b | 0.40±0.16a | 0.27±0.09a |
| Simplicillium | 0.62±0.15b | 0.02±0.01a | 0.04±0.03a |
| Pseudaleuria | 0.53±1.07a | 0.07±0.08a | 1.14±2.20a |
| unclassified_p_Rotifera | 0.51±0.20b | 0.35±0.25ab | 0.05±0.04a |
| unclassified_f_Aspergillaceae | 0.40±0.22a | 2.76±1.36b | 0.03±0.02a |
| Pseudopestalotiopsis | 0.35±0.11a | 1.52±0.69b | 0.11±0.07a |
| Conlarium | 0.32±0.10a | 0.46±0.25a | 0.50±0.13a |
| Talaromyces | 0.24±0.14a | 4.32±3.25b | 0.31±0.146a |
| unclassified_o_Xylariales | 0.21±0.06a | 12.97±25.38a | 0.04±0.04a |
| Pyrenochaetopsis | 0.18±0.06a | 2.18±3.47a | 1.26±0.16a |
| Nakataea | 0.04±0.05a | 0.02±0.03a | 0.51±0.37b |
| unclassified_f_Lasiosphaeriaceae | 0.02±0.02a | 0.08±0.08a | 0.95±0.38b |
| Bipolaris | 0.01±0.02a | 0.07±0.05a | 0.50±0.29b |
| unclassified_f_Mucoraceae | 0.01±0.01a | 0.01±0.01a | 2.22±0.83b |
| Acremonium | 0.00±0.01a | 0.00±0.00a | 0.86±0.04b |
| unclassified_Fungi | 15.54±1.77a | 14.21±7.04a | 7.93±2.99a |

Means are presented with standard deviation (*n* = 4). Different letters indicate significant differences at the P<0.05 level.

**Supplementary Table 8.** Influences of cropping system and cropping time on microbial communities through PERMANOVA analysis (permutational multivariate ANOVA).

| Factors | F. Model | R^2^ | P |
| --- | --- | --- | --- |
| **Bacterial communities** |  |  |  |
| cropping system × cropping time | 18.731 | 0.806 | 0.003 |
| cropping system | 22.734 | 0.695 | 0.002 |
| cropping time | 4.707 | 0.440 | 0.027 |
| **Fungal communities** |  |  |  |
| cropping system × cropping time | 7.060 | 0.611 | 0.001 |
| cropping system | 8.010 | 0.445 | 0.001 |
| cropping time | 3.124 | 0.342 | 0.030 |

**Supplementary Table 9.** The relationships between soil characteristics and soil bacterial and fungal community structures analyzed by the Redundancy analysis (RDA).

| variable | Bacteria | | |  | Fungi | | |
| --- | --- | --- | --- | --- | --- | --- | --- |
|  | RDA1 | r^2^ | p |  | RDA1 | r^2^ | p |
| pH | -0.4868 | 0.4600 | 0.069 |  | 0.7844 | 0.4083 | 0.082 |
| SOC | 0.9839 | 0.9139 | 0.003 |  | -0.3243 | 0.8523 | 0.002 |
| TN | 0.9635 | 0.8089 | 0.003 |  | -0.4069 | 0.8060 | 0.002 |
| NO_3_^-^ | -0.9822 | 0.9493 | 0.001 |  | 0.0303 | 0.6991 | 0.005 |
| NH_4_^+^ | -0.9998 | 0.9790 | 0.001 |  | 0.2074 | 0.8357 | 0.002 |
| AK | -0.9653 | 0.8944 | 0.001 |  | -0.0362 | 0.6066 | 0.006 |
| Olsen-P | 1.0000 | 0.9563 | 0.001 |  | -0.2276 | 0.8360 | 0.002 |
| Lp | 0.7866 | 0.5921 | 0.022 |  | -0.5619 | 0.5470 | 0.016 |
| Hp | 0.9739 | 0.4988 | 0.057 |  | -0.6040 | 0.6006 | 0.012 |

**Supplementary Table 10.** The relationships between environmental factors and soil bacterial and fungal community functions based on the Redundancy analysis (RDA).

| variable | Bacteria | | |  | | Fungi | | |
| --- | --- | --- | --- | --- | --- | --- | --- | --- |
|  | RDA1 | r^2^ | p | |  | RDA1 | r^2^ | p |
| pH | -0.8397 | 0.5019 | 0.003 | |  | 0.6036 | 0.2367 | 0.033 |
| AK | -0.3689 | 0.2046 | 0.081 | |  | 0.6811 | 0.2810 | 0.010 |
| Olsen-P | 0.8422 | 0.4947 | 0.003 | |  | -0.9473 | 0.4747 | 0.002 |
| SOC | 0.9059 | 0.5473 | 0.001 | |  | -0.9727 | 0.4984 | 0.001 |
| TN | 0.9601 | 0.5986 | 0.001 | |  | -0.9569 | 0.4865 | 0.002 |
| NO_3_^-^ | -0.4048 | 0.2211 | 0.063 | |  | 0.7002 | 0.2914 | 0.010 |
| NH_4_^+^ | -0.7126 | 0.3902 | 0.013 | |  | 0.9031 | 0.4383 | 0.004 |
| Lp | 0.9650 | 0.6018 | 0.001 | |  | -0.8877 | 0.4268 | 0.002 |
| Hp | 0.9720 | 0.6111 | 0.001 | |  | -0.8056 | 0.3676 | 0.004 |

**Supplementary Table 11.** Spearman’s rank correlation coefficients and statistical significance between the top 20 phyla of bacteria and fungi to soil properties.

| **Microbes** | **Soil physicochemical properties** | | | | | | | | |
| --- | --- | --- | --- | --- | --- | --- | --- | --- | --- |
|  | **pH** | **AK** | **Olsen-P** | **SOC** | **TN** | **NO_3_^-^** | **NH_4_^+^** | **Lp** | **Hp** |
| **Bacterial phyla** |  |  |  |  |  |  |  |  |  |
| Chloroflexi |  | -0.608* | 0.755** | 0.665* | 0.704* | -0.682* | -0.697* | 0.629* | 0.804** |
| Verrucomicrobiota | 0.691* | 0.727** | -0.608* | -0.737** | -0.697* | 0.650* | 0.625* | -0.741** | -0.664* |
| Firmicutes |  |  | -0.671* |  |  |  |  |  | -0.601* |
| WPS-2 | -0.790** |  |  | 0.874** | 0.907** |  |  | 0.825** | 0.860** |
| Gemmatimonadota | 0.653* |  |  | -0.740** | -0.814** |  |  | -0.720** | -0.846** |
| Myxococcota | 0.737** |  |  | -0.863** | -0.918** |  |  | -0.881** | -0.944** |
| Methylomirabilota | 0.813** |  |  | -0.940** | -0.923** |  |  | -0.928** | -0.876** |
| Desulfobacterota | 0.757** |  |  | -0.901** | -0.941** |  |  | -0.917** | -0.931** |
| Cyanobacteria | -0.756** |  |  | 0.881** | 0.936** |  |  | 0.840** | 0.860** |
| Nitrospirota | 0.720** |  |  | -0863** | -0.889** |  |  | -0.881** | -0.923** |
| Bacteroidota |  | 0.622* | -0.685* |  |  | 0.611* | 0.686* |  |  |
| Dependentiae |  | -0.587* |  |  |  | -0.661* |  |  |  |
| RCP2-54 |  |  |  | -0.679* | -0.743** |  |  | -0.685* | -0.741** |
| **Fungal phyla** |  | | | | | | | | |
| Mortierellomycota | 0.638* | 0.643* | -0.678* | -0.665* | -0.754** | 0.747** | 0.686* | -0.580* | -0.699* |
| Mucoromycota |  | 0.820** | -0.827** |  |  | 0.918** | 0.857** |  |  |
| Rotifera |  | -0.713** | 0.608* |  |  |  |  |  |  |
| Chytridiomycota | -0.646* |  | 0.629* | 0.809** | 0.754** | -0.603* | -0.579* | 0.706* | 0.713** |
| Glomeromycota |  | -0.740** | 0.740** |  |  | -0.824** | -0.743** |  |  |
| Rozellomycota |  | -0.848** | 0.788** | 0618* |  | -0.837** | -0.821** |  |  |
| Calcarisporiellomycota |  | -0.784** | 0.777** |  |  | -0.652* | -.0719** | 0.613* | 0.649* |
| Cercozoa | 0.851** |  |  | -0.821** | -0.834** |  |  | -0.757** | -0.722** |
| Entorrhizomycota | 0.683* | 0.781** | -0.832** | -0.833** | -0.841** | 0.829** | 0.799** | -0.798** | -0.832** |
| Unclassified |  | -0.594* |  | -0.833** |  |  |  |  |  |

Only significant correlations were listed, P≤0.05 and P ≤ 0.01 are marked with “*” and “**”, respectively.
